# Runs of homozygosity reveal signatures of positive selection for reproduction traits in breed and non-breed horses

**DOI:** 10.1186/s12864-015-1977-3

**Published:** 2015-10-09

**Authors:** Julia Metzger, Matthias Karwath, Raul Tonda, Sergi Beltran, Lídia Águeda, Marta Gut, Ivo Glynne Gut, Ottmar Distl

**Affiliations:** Institute for Animal Breeding and Genetics, University of Veterinary Medicine Hannover, Bünteweg 17p, 30559 Hannover, Germany; Lower Saxony State Office for the Environment, Agriculture and Geology, Unit 74, Animal Breeding and Hygiene, Schlossallee 1, 01468 Moritzburg, Germany; Centro Nacional de Análisis Genómico, Parc Científic de Barcelona, Torre I Baldiri Reixac, 4, 08028 Barcelona, Spain

**Keywords:** Runs of homozygosity, Horse population, Selection signature, Reproduction, *KITLG*

## Abstract

**Background:**

Modern horses represent heterogeneous populations specifically selected for appearance and performance. Genomic regions under high selective pressure show characteristic runs of homozygosity (ROH) which represent a low genetic diversity. This study aims at detecting the number and functional distribution of ROHs in different horse populations using next generation sequencing data.

**Methods:**

Next generation sequencing was performed for two Sorraia, one Dülmen Horse, one Arabian, one Saxon-Thuringian Heavy Warmblood, one Thoroughbred and four Hanoverian. After quality control reads were mapped to the reference genome EquCab2.70. ROH detection was performed using PLINK, version 1.07 for a trimmed dataset with 11,325,777 SNPs and a mean read depth of 12. Stretches with homozygous genotypes of >40 kb as well as >400 kb were defined as ROHs. SNPs within consensus ROHs were tested for neutrality. Functional classification was done for genes annotated within ROHs using PANTHER gene list analysis and functional variants were tested for their distribution among breed or non-breed groups.

**Results:**

ROH detection was performed using whole genome sequences of ten horses of six populations representing various breed types and non-breed horses. In total, an average number of 3492 ROHs were detected in windows of a minimum of 50 consecutive homozygous SNPs and an average number of 292 ROHs in windows of 500 consecutive homozygous SNPs. Functional analyses of private ROHs in each horse revealed a high frequency of genes affecting cellular, metabolic, developmental, immune system and reproduction processes. In non-breed horses, 198 ROHs in 50-SNP windows and seven ROHs in 500-SNP windows showed an enrichment of genes involved in reproduction, embryonic development, energy metabolism, muscle and cardiac development whereas all seven breed horses revealed only three common ROHs in 50-SNP windows harboring the fertility-related gene *YES1*. In the Hanoverian, a total of 18 private ROHs could be shown to be located in the region of genes potentially involved in neurologic control, signaling, glycogen balance and reproduction. Comparative analysis of homozygous stretches common in all ten horses displayed three ROHs which were all located in the region of *KITLG,* the ligand of *KIT* known to be involved in melanogenesis, haematopoiesis and gametogenesis.

**Conclusions:**

The results of this study give a comprehensive insight into the frequency and number of ROHs in various horses and their potential influence on population diversity and selection pressures. Comparisons of breed and non-breed horses suggest a significant artificial as well as natural selection pressure on reproduction performance in all types of horse populations.

**Electronic supplementary material:**

The online version of this article (doi:10.1186/s12864-015-1977-3) contains supplementary material, which is available to authorized users.

## Background

The modern horse population represents a particularly heterogenous group influenced over the time by various selective pressures [1]. However, in studies on genetic diversity a contrasting homogeneity within breeds or non-breeds has been observed [2–4]. In particular breeds like the Arabian, Hanoverian and Saxon-Thuringian Heavy Warmblood have been shaped by intense human selection for specific abilities and characteristics that meet with requirements for optimal performance whereas environmental conditions have particularly influenced non-breed horses. The Dülmen Horse and the Sorraia can be characterized as non-breeds with a robust constitution for primitive living conditions not subjected to human selection criteria for specific breeding aims but to natural selection [5–7]. Strong selective pressures result in a reduction of genetic diversity which is characterized by long stretches of consecutive homozygous genotypes in the genome known as runs of homozygosity (ROH) [8–11]. Size and frequency of ROHs give evidence for relatedness within and in-between populations.

In horses, signals of selection have been investigated in 744 horses of 33 breeds using whole-genome single nucleotide polymorphism (SNP) array data [1]. Potential genomic targets of selection were observed within breeds by F_ST_-based statistics in 500-kb windows and revealed common haplotypes in the region of coat color genes, size and performance traits. The highest signature of selection was found in the Paint and Quarter Horse on ECA18 in the region of the *myostatin* gene (*MSTN*). Positively selected loci for performance have also been detected in a Thoroughbred population study based on microsatellite markers [12]. Candidate regions for exercise adaption including fatty acid oxidation, increased insulin sensitivity and muscle strength have been suggested as potential selection targets.

Signals of selection have also been investigated in other mammals including cattle, dog, pig and human, frequently scanning ROHs as diversity indices [1, 9–11, 13]. In human, ROHs were considered valuable for population demographic analyses and allowed reliable differentiation of human indigenous populations from distinct continents [14]. Shorter homozygous stretches helped to characterize population specific properties whereas ROH longer than 0.5 Mb could be shown to be frequent in all populations [15, 16]. It was proposed that ROHs longer than 1 Mb were quite more common in outbred individuals. In addition to population genetic applications, ROH detection was suggested to be valuable for mapping of causative mutations for recessive diseases [17, 18]. A study for schizophrenia identified nine risk ROHs which were significantly more frequent in affected patients and harbored disease-associated genes [19].

In domestic animals, especially performance related phenotypes and breed specific characteristics were mainly in focus of ROH analyses [9, 20]. A whole-genome comparative detection of ROHs in a sliding window approach was applied for pigs in wild and domesticated populations [9]. Two overlapping ROHs were identified in the European breeds harboring genes involved in cell differentiation. In Large White and Landrace pigs an exclusive ROH could be shown to be located in the region of the growth related *pleiomorphic adenoma gene 1* (*PLAG1)*. Further breed specific ROH analyses for Chinese and Western pigs revealed loci under selection important for high-altitude adaption in Tibetan pigs as well as a coat color locus in the region of *endothelin receptor type B* (*EDNRB)* in Chinese belted pigs [21].

Signatures of selection affecting coat color and body size traits could also be observed in genome-wide ROH scans for dogs [22]. It was suggested that ancestral genetic variations were transformed into specific characteristics of different dog breeds [13, 22]. Next generation sequencing (NGS) data from dogs and wolves revealed regions of potential selection in domesticated dogs which affect metabolism and thus suggest a potential adaption to starch digestion [13, 23]. In the Lundehund, fifteen regions with long-range haplotypes indicated potential signatures of positive selection for polydactyly, body size and male fertility [24]. In cattle, a large number of ROHs have been shown to be widely distributed among various breeds and demonstrated its utility for prediction of inbreeding coefficients and relatedness [10, 25, 26]. Haplotype-frequency based approaches revealed signatures of selection in the region of genes affecting reproduction and muscle formation [27]. A genome-wide scan in Holstein cattle identified milk yield, composition, reproduction and behavioral traits in potentially selected regions [28]. Similar observations were made in an U.S. Holstein cattle study which investigated the distribution of ROHs in different milk production groups [29]. Forty genomic regions in potential signatures of selection were identified in SNP array data harboring loci for milk, fat and protein yield. However, the use of SNP arrays for ROH detection was suggested to be limited mainly for low SNP density reasons [27, 28, 30]. Higher resolution genomic analyses on basis of whole-genome data enabled the use of 15 million SNPs from 43 Fleckvieh cattle for powerful detection of selected traits [20]. Candidate regions for coat color, neurobehavioral functioning and sensory perception were found in ROH regions suggesting domestication-related signatures of selection. The accuracy of ROH detection in NGS data was shown to be high if corrected for bias by hidden errors in genotyping data [31].

In this study, whole-genome sequences of ten horses were used for analysis of ROHs in a sliding window approach. 50-SNP and 500-SNP windows were chosen for reliable detection of ROHs of different sizes. ROHs exclusively found in individual horses or breeds were further investigated for their gene content potentially affected by targeted selection for specific appearance and function.

## Results

### Sequencing and variant detection

Whole-genome sequences of a Dülmen Horse, two Sorraia, an Arabian, a Saxon-Thuringian Heavy Warmblood descendent from the Old-Oldenburg breed, a Thoroughbred and four Hanoverian were obtained using NGS. Mapping to the reference genome EquCab2.70 resulted in a mean coverage of 19.90X for the Dülmen Horse, 17.34X and 17.55X for the two Sorraia, 19.14X for the Arabian, 20.06X for the Saxon-Thuringian Heavy Warmblood, 5.92X for the Thoroughbred and 15.66X-35.18X for the four Hanoverian (Additional file 1). Raw data of variant detection revealed 3,865,613-7,147,081 SNPs and 698,724-992,338 insertions/deletions (INDELs) in each of the ten horses. After stringent quality control, a total of 11,325,777 SNPs were filtered out and used for ROH analysis. The mean heterozygosity for these SNPs per site was 0.28 in the Dülmen Horse and Arabian, 0.24 and 0.25 in the Sorraia horse, 0.29 in the Saxon-Thuringian Heavy Warmblood, 0.29 in the four Hanoverian and 0.21 in the Thoroughbred.

### Sequence error estimation

We estimated sequence errors on the basis of SNP50 BeadChip data in five horses. The results of BeadChip analysis were assumed to be error free. The rate of false-negative SNPs was calculated based on heterozygous SNPs in BeadChip data which were homozygous in NGS data. We detected false-negative rates of 0.24–0.26 in the four Hanoverian and 0.22 in the Arabian (Table 1). The false-positive rate was at 3.8 x 10^−4^ to 9.8 × 10^−4^using all SNP positions of the BeadChip data. More stringent error estimations in long SNPChip ROH regions of >10 Mb compared to filtered NGS sequence revealed even lower error rates at 3.1 × 10^−4^ to 5.9 × 10^−5^.

**Table 1:** Sequence error estimation

Number of SNPs	Horse 4	Horse 5	Horse 6	Horse 7	Horse 9
	Hanoverian	Hanoverian	Hanoverian	Hanoverian	Arabian
Heterozygous in NGS data	891,514	815,810	833,149	832,549	812,952
Heterozygous in BeadChip data	16,426	15,469	15,873	15,987	14,008
Homozygous in BeadChip data	33,403	34,360	33,956	33,842	35,821
Heterozygous in NGS and BeadChip data	3,555 (7.14 %)	3,239 (6.50 %)	3,306 (6.64 %)	3,396 (6.82 %)	2,960 (5.94 %)
Heterozygous in BeadChip and homozygous in NGS data	12,867 (25.83 %)	12,223 (24.54 %)	12,565 (25.22 %)	12,589 (25.27 %)	11,047 (22.17 %)
(False-negative rate)					
Homozygous in BeadChip and heterozygous in NGS data	19 (3.8 × 10^−4^of all 49,813 positions validated in BeadChip)	29 (5.8 × 10^−4^of all 49,813 positions validated in BeadChip)	38 (7.6 × 10^−4^of all 49,815 positions validated in BeadChip)	49 (9.8 × 10^−4^of all 49,823 positions validated in BeadChip)	28 (5.6 × 10^−4^of all 49,824 positions validated in BeadChip)
(False-positive rate)					
Homozygous in >10 Mb BeadChip ROH regions and heterozygous in NGS data	5.9 × 10^−5^	3.8 × 10^−5^	2.2 × 10^−5^	3.1 × 10^−4^	2.6 × 10^−5^
(Robust false-positive rate)					

Number of detected Next Generation sequencing (NGS) -SNPs and their sequence error rates in comparison to Illumina SNP50 BeadChip data. The BeadChip data are assumed to be error free

### ROH detection

**Fig. 1:**
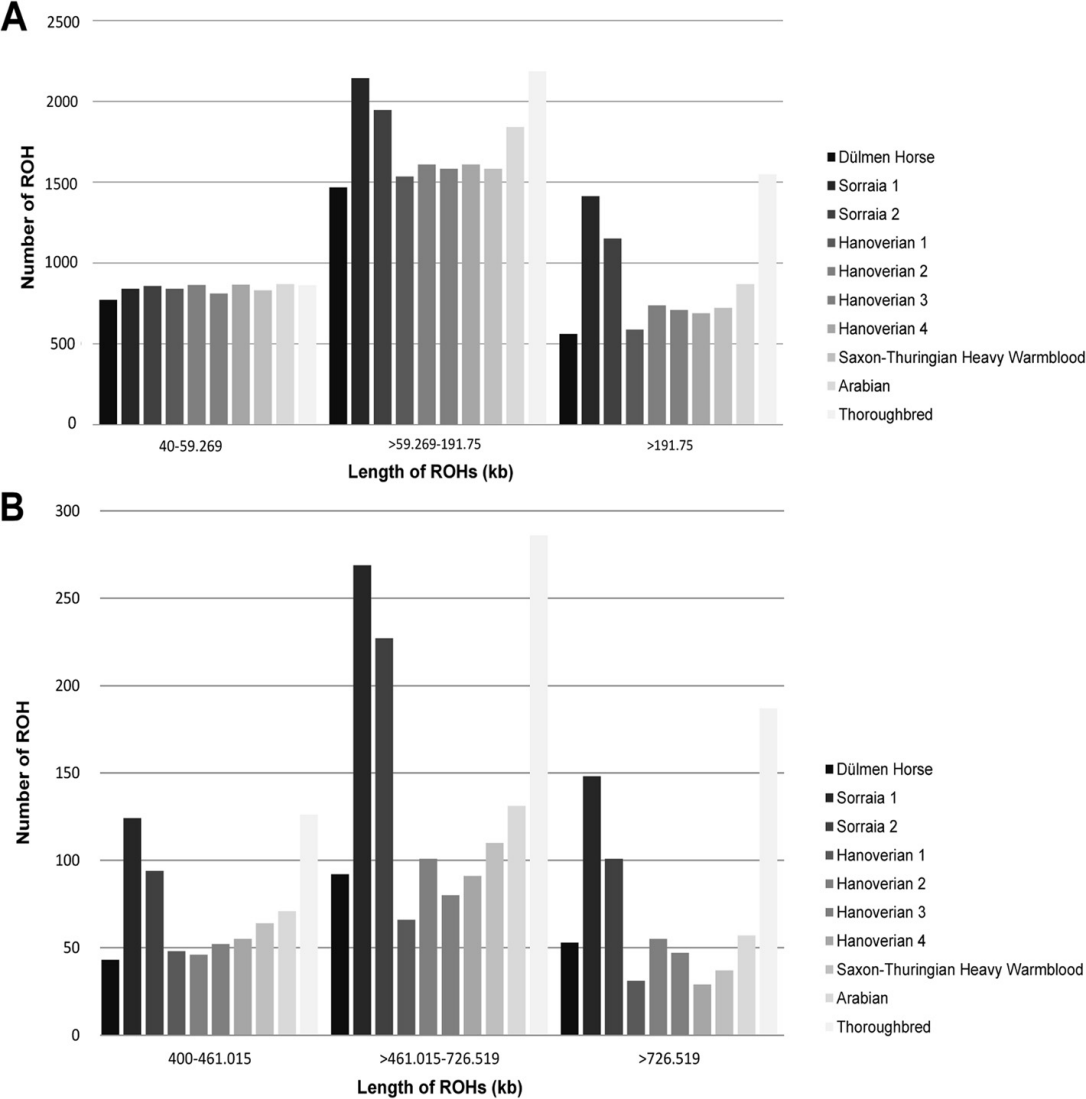
Number and size of runs of homozygosity (ROH) detected in ten horses. The length of ROHs was categorized into small, medium and large ROH regions. The results of plink analysis with windows of minimum amount of 50 homozygous SNPs (**a**) and 500 homozygous SNPs (**b**) are shown

An average number of 3492 ROHs was detected for the ten horses in windows of minimum amount of 50 homozygous SNPs and an average number of 292 ROHs in windows of 500 homozygous SNPs (Table 2). The number of smaller ROHs of 40–59 kb was almost equally distributed in all ten horses, whereas ROHs >59 kb were comparatively high in the two Sorraia horses and in the thoroughbred (Fig. 1). ROH detection in larger windows of >400 kb revealed even a more distinct distribution, showing ROHs particularly frequent in the Sorraia and Thoroughbred but also in the Arabian. As indicated by the number and size of ROH, the total length of ROHs in sliding windows of at least 50 SNPs was notably high in the Thoroughbred (953 Mb) and the two Sorraia (867 and 730 Mb) and comparatively high in the Arabian (566 Mb, Additional file 2). The F_ROH_ estimated for 50-SNP windows were 0.43 in the Thoroughbred, 0.39 and 0.33 in the Sorraia horses, 0.25 in the Arabian. The four Hanoverian as well as the Saxon-Thuringian Heavy Warmblood and the Dülmen Horse showed F_ROH_ranging from 0.18 to 0.22. Similar distributions of F_ROH_ could be observed for 500-SNP windows, showing the highest values of 0.18 in the Thoroughbred as well as 0.16 and 0.12 in the Sorraia horses.

**Table 2:** Summary of runs of homozygosity (ROHs) detected in whole genome sequencing data of ten horses

	Horse 1	Horse 2	Horse 3	Horse 4	Horse 5	Horse 6	Horse 7	Horse 8	Horse 9	Horse 10
	Dülmen	Sorraia	Sorraia	Hanoverian	Hanoverian	Hanoverian	Hanoverian	Saxon-Thuringian Heavy Warmblood	Arabian	Thoroughbred (SRR1055837)
Number of ROHs (50)	2804	4395	3955	2965	3214	3104	3167	3138	3581	4595
Number of private ROHs (50)	722	1578	1349	538	752	662	709	739	934	1940
Number of ROHs (500)	188	541	422	145	202	179	175	211	259	599
Number of private ROHs (500)	152	352	285	80	150	84	109	123	153	459

The number of ROHs in windows of a minimum amount of 50 homozygous SNPs and 500 homozygous SNPs are shown. All ROHs were compared in-between horses and filtered for private ROH regions that could only be found in one horse

50 = A minimum amount of 50 homozygous SNPs

500 = A minimum amount of 500 homozygous SNPs

### Private ROHs and functional annotation

Functional annotation of genes located in private horse specific ROH regions, which could not be detected in one of the other horses under analysis, was performed in order to get an insight into biological processes affected by genes in horse specific homozygous segments. PANTHER gene list analysis for 50-SNP as well as 500-SNP windows revealed a high percentage of genes involved in cellular processes (GO:0009987), metabolic processes (GO:0008152) as well as biological regulations (GO:0065007), localization (GO:0051179) and developmental processes (GO:0032502) in all private ROHs of the analyzed ten horses (Table 3, Additional file 3). Further rates of gene hits affecting responses to stimulus (GO:0050896), cellular processes (GO:0071840; GO:0032501), immune system processes (GO:0002376), apoptotic processes (GO:0006915), biological adhesion (GO:0022610) and reproduction (GO:0000003) could also be observed in annotation results.

**Table 3:** Functional annotations in private runs of homozygosity (ROH) of 50-SNP windows

PANTHER gene ontology terms	Horse 1	Horse 2	Horse 3	Horse 4	Horse 5	Horse 6	Horse 7	Horse 8	Horse	Horse 10
	Dülmen	Sorraia (%)	Sorraia (%)	Hanoverian (%)	Hanoverian (%)	Hanoverian (%)	Hanoverian (%)	Saxon-Thuringian Heavy Warmblood (%)	Arabian (%)	Thoroughbred (SRR1055837) (%)
	Horse (%)									
Cellular component organization or biogenesis (GO:0071840)	3.6	3.1	2.8	3.2	4.0	4.5	5.3	4.4	3.5	4.0
Cellular process (GO:0009987)	18.7	19.9	18.2	18.8	21.5	20.2	20.3	20.8	19.3	18.6
Localization (GO:0051179)	8.3	9.3	10.2	10.3	7.5	11.3	9.9	11.2	10.6	8.6
Apoptotic process (GO:0006915)	2.2	1.5	2.8	2.1	1.7	1.9	1.8	1.4	2.7	2.6
Reproduction (GO:0000003)	1.9	1.8	3.7	2.1	1.3	2.0	0.7	1.2	0.4	1.8
Biological regulation (GO:0065007)	10.2	9.3	9.2	9.8	8.1	9.7	11.3	9.1	9.7	9.6
Response to stimulus (GO:0050896)	5.2	5.6	6.3	5	4.5	4.6	3.7	4.7	5.2	6.3
Developmental process (GO:0032502)	8.9	6.8	8.2	10.1	11.1	9.7	8.5	6.8	8.9	8.6
Multicellular organismal process (GO:0032501)	5.6	6.1	5.4	7.4	5.5	6.0	6.2	5.9	5.6	5.4
Locomotion (GO:0040011)	0.1	0.0	0.0	0.0	0.0	0.0	0.0	0.0	0.4	0.0
Biological adhesion (GO:0022610)	2.8	4.2	2.4	3.7	6.8	2.8	3.2	3.5	2.7	3.1
Metabolic process (GO:0008152)	27	26.8	24.2	21.8	23.4	22.7	24.7	25.3	26.3	23.4
Growth (GO:0040007)	0.1	0.0	0.0	0.0	0.0	0.0	0.0	0.0	0.0	0.0
Immune system process (GO:0002376)	5.4	5.5	6.6	5.6	4.5	4.5	4.2	5.6	4.8	8.2

PANTHER gene list analysis (http://www.pantherdb.org/) was performed for genes in private ROH regions which could be exclusively found in one specific horse. The percent of gene hits against total number of process hits involved in specific biological processes are shown

**Fig. 2:**
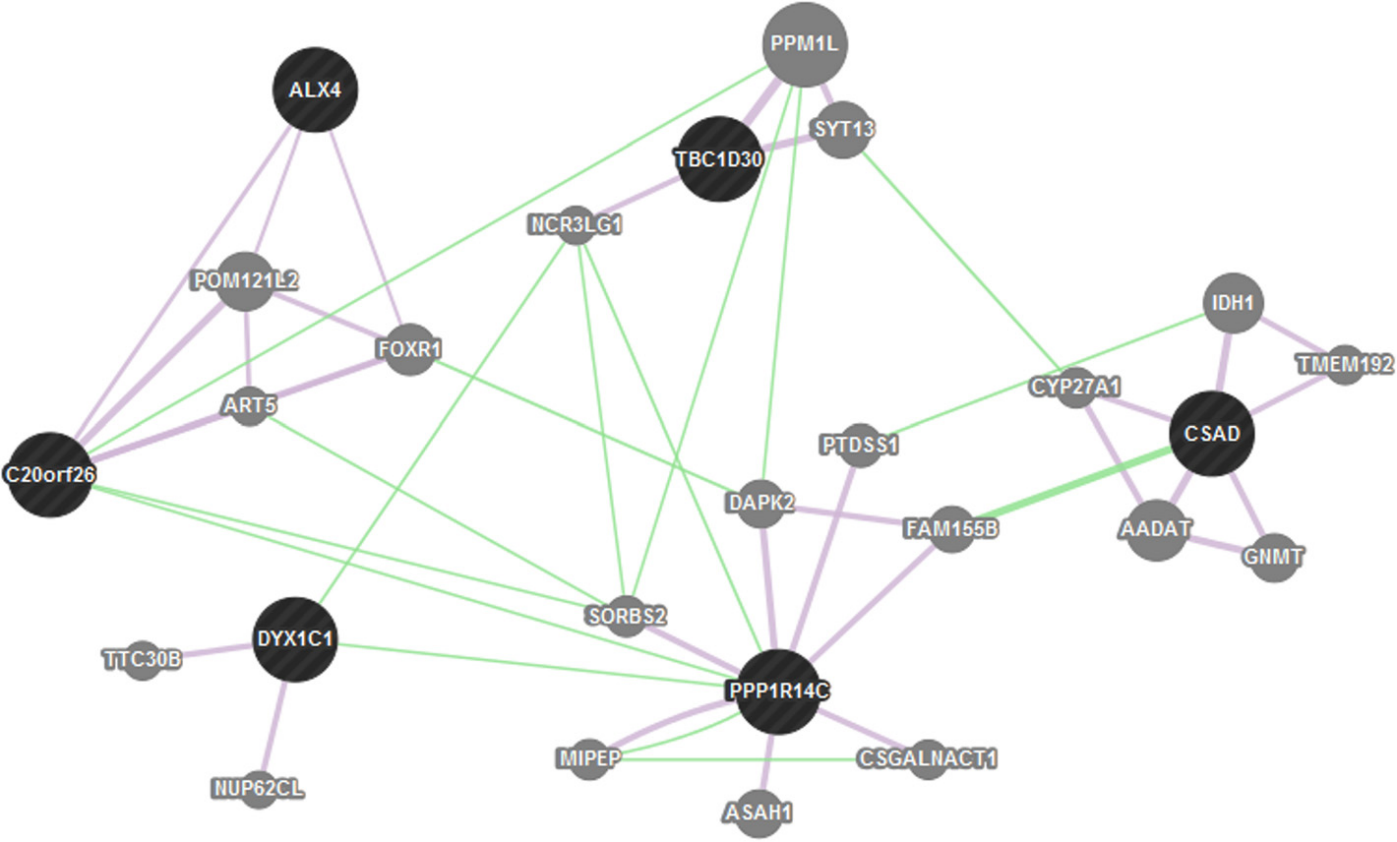
GeneMANIA network of six genes in ROH regions shared by the Hanoverian. The genes of interest are represented as black circles, related genes as grey circles. Genetic interactions are displayed as green lines and co-expressions as violet lines. All six genes are interrelated with each other

**Fig. 3:**
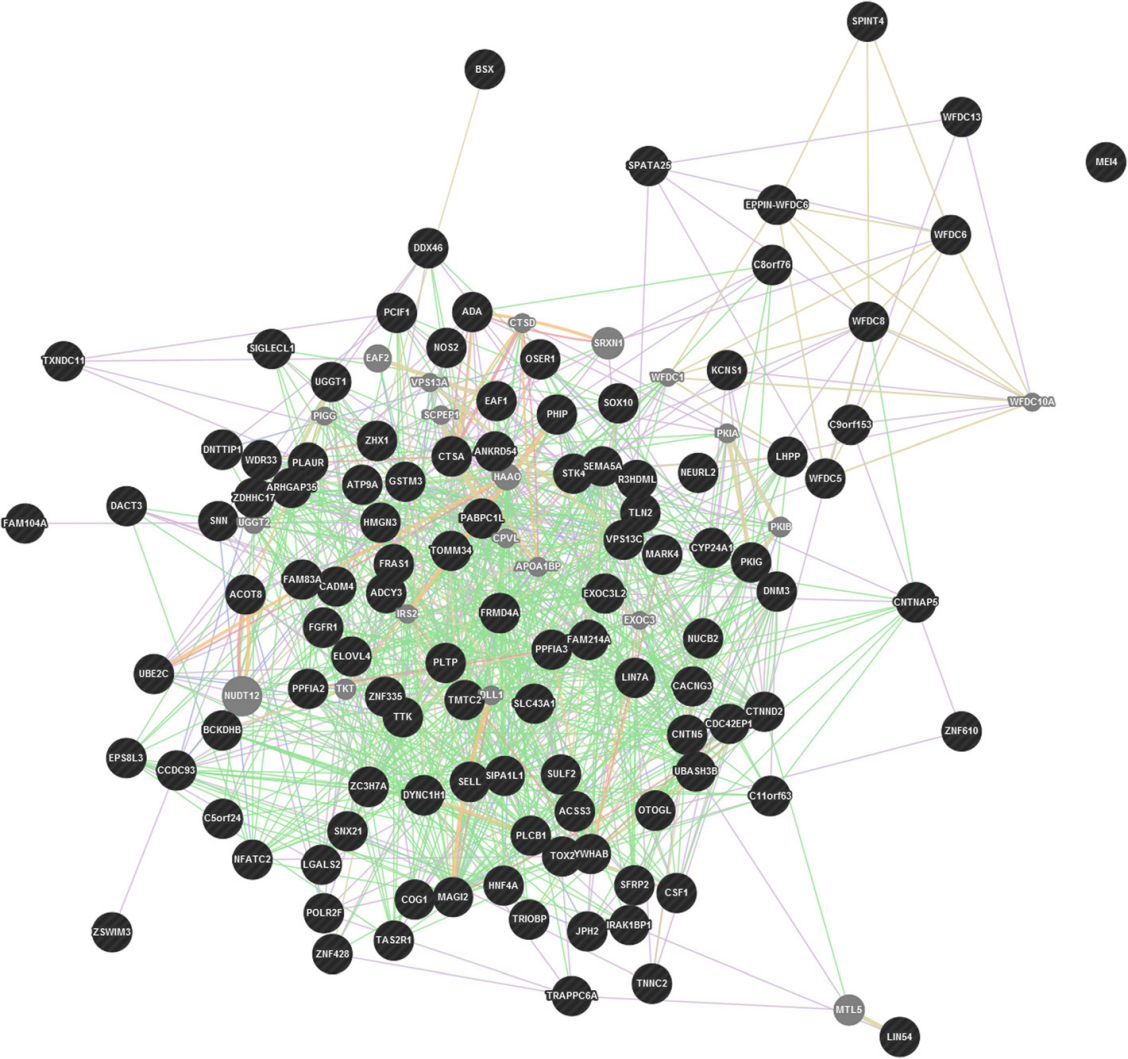
GeneMANIA network of 139 genes in 50-SNP window ROH regions shared by non-breed horses. The genes of interest are represented as black circles, related genes as grey circles. Genetic interactions are displayed as light green lines, predicted related genes as orange lines, physical interactions as red lines, co-localization as blue lines, shared protein domains as dark green lines and co-expressions as violet lines

**Fig. 4:**
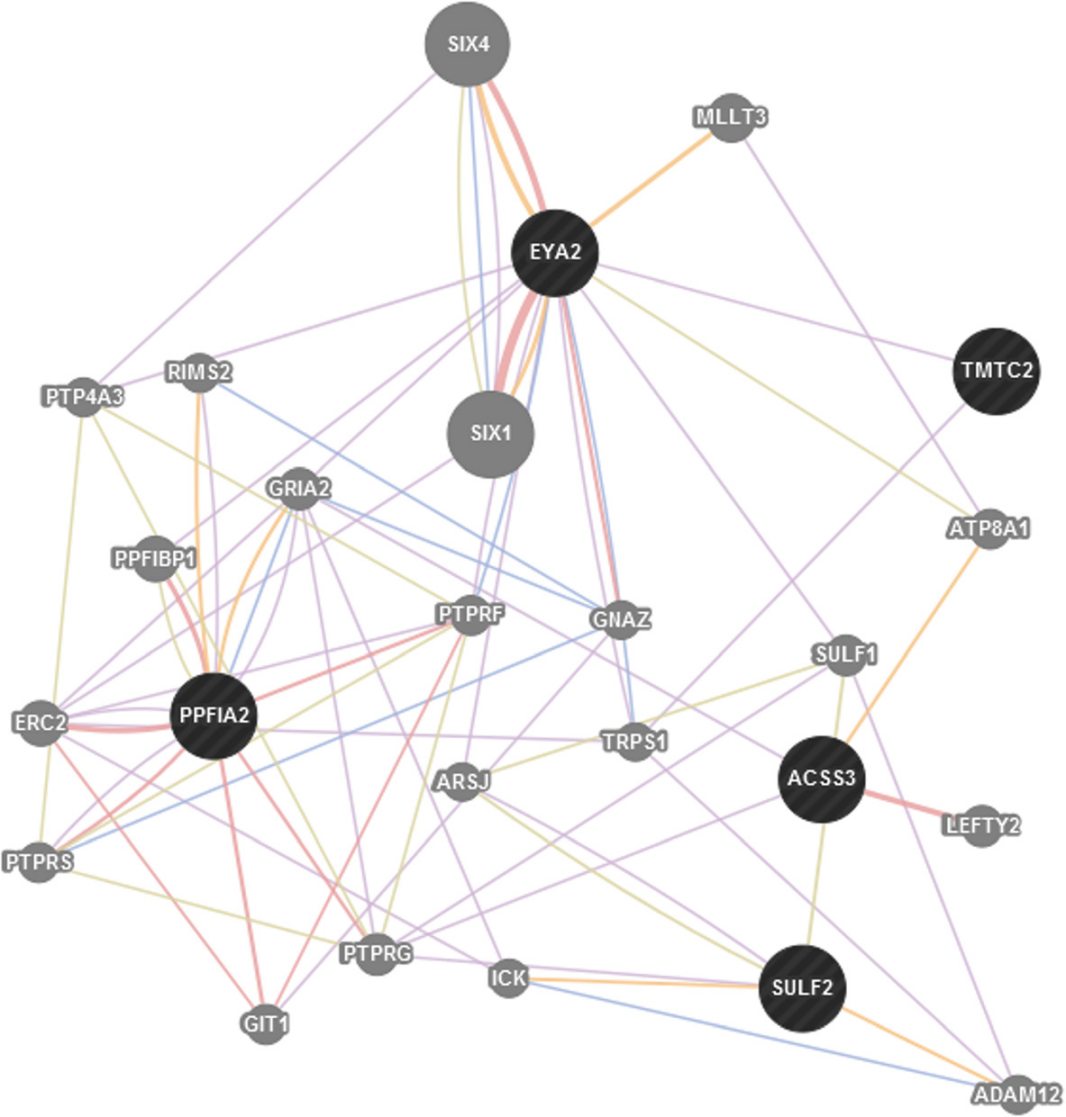
GeneMANIA network of 7 genes in 500-SNP window ROH regions shared by non-breed horses. The genes of interest are represented as black circles, related genes as grey circles. Predicted related genes are displayed as orange lines, physical interactions as red lines, co-localization as blue lines, shared protein domains as dark green lines and co-expressions as violet lines

Analysis of shared private ROHs in specific breed horses revealed 18 ROHs common in all four Hanoverian but not in the other analyzed horses in 50-SNP windows and no shared ROHs in 500-SNP windows (Table 4). The 18 ROHs contained four novel genes and six genes known as *dyslexia susceptibility 1 candidate 1 (DYX1C1), protein phosphatase 1, regulatory (inhibitor) subunit 14C (PPP1R14C), cilia and flagella associated protein 61 (CFAP61/C20orf26), cysteine sulfinic acid decarboxylase (CSAD), TBC1 domain family, member 30 (TBC1D30)* and *ALX homeobox 4 (ALX4),* which were shown to be related by direct genetic interactions or co-expression (Fig. 2). A dense network of genetic interactions could also be found in-between genes located in private ROHs exclusively found in the non-breed horses Dülmen Horse and Sorraia (Fig. 3 and 4). In total, 198 ROHs could be detected in 50-SNP windows covering 139 genes (Additional file 4). The largest ROHs for non-breed horses of 324,707-163,116 base pairs were located in the region of the developmental and signaling genes *secreted frizzled-related protein 2 (SFRP2), fraser extracellular matrix complex subunit 1 (FRAS1), interleukin-1 receptor-associated kinase 1 binding protein 1 (IRAK1BP1), pleckstrin homology domain interacting protein (PHIP), acyl-CoA synthetase short-chain family member 3 (ACSS3), protein tyrosine phosphatase, receptor type, f polypeptide, interacting protein (liprin), alpha 2 (PPFIA2)* and did also cover a gene-rich region which included *spermatogenesis associated 25 (SPATA25), acyl-CoA thioesterase 8 (ACOT8)* and *troponin C type 2 fast (TNNC2)*. ROH detection in 500-SNP windows revealed seven common ROH regions for non-breed horses. They were located on horse chromosomes 22 and 28 in or near by ROHs already found in 50-SNP windows. The largest region showed a size of 576,454 base pairs*.* In contrast to non-breeds, the whole group of breed horses (Hanoverian, Arabian, Saxon-Thuringian Heavy Warmblood and Thoroughbred) revealed only three common ROHs in 50-SNP windows and no common ROHs in 500-SNP windows (Additional file 5). The largest private ROH with 54,740 base pairs shared by all eight breed horses revealed a Tajima’s D of −1.0 and could be shown to harbor the *V-Yes-1 Yamaguchi Sarcoma Viral Oncogene Homolog 1 (YES1).*

**Table 4:** Shared private runs of homozygosity (ROH) in 50-SNP windows

ECA	Position	Number of SNPs in shared parts of ROH regions	Size of shared ROH (bp)	Gene ID	Human ortholog	Gene name/function
1	135530600-135573693	192	43094	ENSECAG00000010951, ENSECAG00000001112, ENSECAG00000004438, ENSECAG00000001272	*DYX1C1, novel gene, novel gene, novel gene*	*dyslexia susceptibility 1 candidate 1*
4	13434735-13434933	17	199	-	-	*-*
4	16813531-16813643	4	113	-	*-*	*-*
4	48740665-48745235	7	4571	-	*-*	*-*
6	70162066-70181092	35	19027	ENSECAG00000006878	*CSAD*	*cysteine sulfinic acid decarboxylase*
6	80540783-80586091	227	45309	ENSECAG00000024798	*TBC1D30*	*TBC1 domain family, member 30*
6	81785611-81786700	28	1090	-	*-*	*-*
8	50498336-50582995	392	84660	-	*-*	*-*
12	9146140-9204195	70	58056	ENSECAG00000019892	*ALX4*	*ALX homeobox 4*
14	30412967-30412967	1	1	-	*-*	*-*
14	85610289-85611904	69	1616	-	*-*	*-*
15	5973552-5973559	3	8	-	*-*	*-*
15	79802636-79806388	119	3753	-	*-*	*-*
16	23766626-23821105	311	54480	ENSECAG00000002081	*novel gene*	
22	4841367-4841408	3	42	ENSECAG00000004364	*CFAP61*	*cilia and flagella associated protein 61*
22	16310734-16310750	3	17	-	*-*	*-*
28	39200676-39218496	29	17821	-	*-*	*-*
31	16634998-16711978	347	76981	ENSECAG00000023857	*PPP1R14C*	*protein phosphatase 1, regulatory (inhibitor) subunit 14C*

The consensus ROH regions and genes in private ROH regions shared in four Hanoverian are shown. The number of SNPs and size of shared ROH indicate the overlap of homozygous variants

**Fig. 5:**
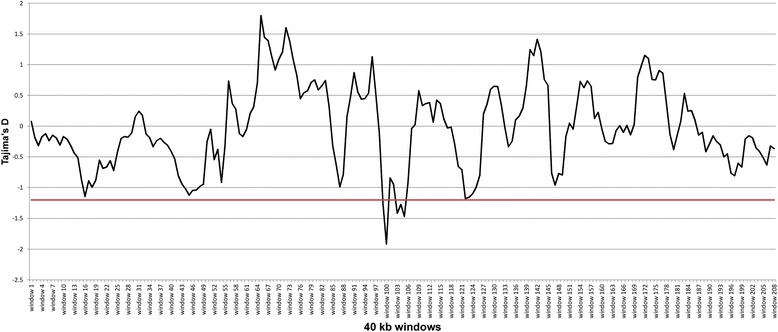
Tajima’s D estimate on equine chromosome 28 in the region of 13.68–15.75 Mb for all ten horses. Decreased Tajima’s D values below −1.2 can be observed in the consensus ROH extending over 14.65–14.78 Mb and harboring KITLG

Evaluations of consensus ROHs for all ten horses revealed three ROHs which were all located on chromosome 28 at 14,656,676–14,778,472 Mb in the region of *KIT ligand (KITLG,* Table 5). No common ROHs could be found in 500-SNP windows in all ten horses. Tajima’s D test statistics confirmed a deviation from neutrality in this region showing values below −1.2 in windows covering 14.65–14.78 Mb (Fig. 5, Additional file 6).

**Table 5:** Shared runs of homozygosity (ROH) in 50-SNP windows

ECA	Position	Number of SNPs in shared parts of ROH regions	Size of shared ROH (bp)	Gene ID	Human ortholog	Gene name/function
28	14656676-14778472	310	121797	ENSECAT00000000221	*KITLG*	*KIT ligand*
28	14781594-14843608	123	62015			
28	14844608-14951801	175	107194			

The consensus ROH regions and genes in all ten horses are shown. The number of SNPs and size of shared ROH indicate the overlap of homozygous variants

### Functional variations in ROH regions

Private ROHs were further investigated for variants which might have a functional impact on horse group specific traits. In non-breed horses, 166 mutations with predicted high or moderate effects within ROHs of 50-SNP windows and 5 mutations within ROHs of 500-SNP windows could be filtered out (Additional file 7 and 8). Three SNPs located on chromosome 10 at 19,334,666 (p.Val667Leu), 34,179,092 (p.Asp5Asn) and 34,221,357 Mb (p.Val208Ile) and one SNP on chromosome 28 at 8,441,975 Mb (p.Met260Thr) were found homozygous for the mutated allele in the Dülmen Horse and the two Sorraia horses but heterozygous or homozygous wild type in all breed horses. The Val667Leu variant in exon 10 of *Histidine Rich Calcium Binding Protein (HRC)* was predicted to be deleterious (SIFT score 0.01) whereas the other three variants located in *Elongation Of Very Long Chain Fatty Acids Protein 4 (ELOVL4), Phosphotyrosine Picked Threonine-Protein Kinase (TTK)* and *ACSS3* were proposed to be tolerated (SIFT score 0.32, 0.34, 0.12). In contrast to non-breeds, breed horses harbored no variants with high or moderate effects in private ROHs. Nevertheless, the four Hanoverians could be shown to harbor four SNPs in their private ROH regions in the genes *DYX1C1, CSAD* and in the novel gene *ENSECAG00000004438* (Additional file 9). These missense mutations showed no specific genotypes which could be exclusively found in the four Hanoverians. Furthermore, a closer examination of the consensus ROHs of all ten horses revealed a total of seven SNPs in the intronic region of *KITLG* but no variants with high or moderate effects.

## Discussion

The detection of ROHs in ten horses of six different populations allowed us to estimate the genetic diversity in breeds or non-breeds and their signatures of potential selection. Smaller ROHs could be found in all horses to a very high number whereas ROHs of a larger size >59 kb and also longer stretches of consecutive homozygous genotypes >400 kb showed quite distinct distribution among different horse populations. Long homozygous stretches and consequently high inbreeding coefficients characterized the Sorraia and Thoroughbred, which were shown to be closed populations, as well as the Arabian derived from a relatively narrow genetic base [32–34]. Especially in the Thoroughbred the low genetic diversity was supposed to be a result of high selective pressures for specific traits of racing performance [12].

In contrast, the four Hanoverian sport horses in our study showed a low number of ROHs and relatively low values for F_ROH_ indicative for inbreeding. Nevertheless, they shared 18 ROHs which harbored six genes potentially important for appearance and performance in sport horses. One of these genes, the homeodomain transcription factor coding gene *ALX4* was proposed to play an essential role in the skeletal mineralization and epidermal development in human and mice [35, 36]. Neurologic activity could be shown to be affected by *CSAD,* regulating intracellular calcium levels in neurons by its influence on taurine biosynthesis, and *DYX1C1* involved in neuronal migration [37–39]. The candidate gene *TBC1D30* has been characterized as a signal transducing peptide [40]. Comparative analyses of indicine and taurine cattle revealed signatures of selection and copy number variations in the region of *TBC1D30* [41]. In *KEPI (PPP1R14C)*-knockout mice, a reduce response to repeated morphine injections suggested an important role of *KEPI* in the regulation of analgesic tolerance [42]. *KEPI* was shown to be expressed in brain regions of drug reward, locomotor control and nociception [42, 43]. Furthermore, it was supposed to play an important role for the regulation of glycogen synthase by its inhibitive effect on protein phosphatase 1 (PP1) [44]. A significant impact on fertility could be observed in association with *CFAP61* which was shown to affect cilia and flagella motility [45, 46]. It can be assumed that these functional effects on neurologic control, signaling pathways, glycogen balance and reproduction might represent important targets of selection for the Hanoverian, which has become a specifically shaped breed into a modern sport horse type. In comparison to ROH analysis of all breed horses, the number of ROHs in the Hanoverian was relatively high probably as a result of breed specific similarities. However, despite significant differences in-between breeds, the whole group of breed horses revealed a region of potential selection harboring a fertility-related gene. *YES1* could be shown to be an essential protein tyrosine kinase for self-defensive mechanisms in spermatocytes [47]. During testicular heat stress a significantly upregulated expression of *YES1* was supposed to antagonize apoptotic processes to maintain spermatogenic differentiation and male fertility. In addition to that, it was even more intriguing that ROH analyses in non-breed horses also suggested a high positive selection for reproduction in mainly naturally selected horses. One of the largest ROHs could be shown to harbor the fertility related gene *SPATA25* which is known to be mainly expressed in testis in human. Studies of obstructive azoospermia revealed a significantly reduced expression level in affected patients in comparison to fertile persons [48]. Other candidate genes were proposed to be involved in embryonic development. Analyses of *FRAS1* deficient mice revealed phenotypic defects affecting embryonic epithelial basement membranes and internal organs [49]. Furthermore a number of genes involved in energy metabolism *(Acyl-coenzyme A synthetase 3, ACSS3; thioesterase 8, ACOT8)* [50, 51] and muscle development *(NEURL2)* [52, 53] could be found in large non-breed specific ROHs. The differentiation and survival of cardiomyocytes was supposed to be affected by *SFRP2* [54]. It was shown that *SFRP2* plays an important role in myocardial survival and is involved in ischemic injury repair of cardiomyocytes. The assumption of a potential non-breed specific effect on myocardial regulation for greater endurance in free range conditions was supported through the detection of a functional variant with a possibly deleterious impact on *HRC*. It was suggested that different expression levels of HRC can affect CA^2+^ homeostasis and contractile function of the heart [55, 56]. In human and mice affected with heart failures, the HRC expression levels could be shown to be significantly decreased. In conclusion, we propose that non-breed horses underlie a selection mainly driven by nature which affects reproduction, embryonic development, energy metabolism and cardiac development traits. These results confirm the suggestion that metabolic processes and morphogenesis play an important role for survival and maintenance in non-breeds [57].

Despite the specific genetic features in non-breeds as well as breeds and the general differences in the number and length of ROHs in various horse breeds, a functional enrichment of genes affecting cellular, metabolic and developmental as well as immune system and reproduction processes could be shown in ROHs in all ten horses.

These results suggest that despite the low number of individuals in some breeds or non-breeds these ten horses presumably represent a general phenomenon in horse populations. We assume that regions of genes involved in fundamental processes essential for development and sustainment of individuals and populations underlie high selective pressures and accordingly limited variations. A main focus which could be found in all breeds, specific breeds (Hanoverian) and also in non-breeds was a potential selection for traits of reproduction. Essential genes for processes affecting fertility, embryonic development and birth varied among different horse populations but could be assumed to play a key role in artificial or natural selection as well. Reproduction performance has been shown to be of high economic importance in breeds and of vital importance for non-breeds to ensure survival in the wild [5, 58]. Various studies in livestock came to the same conclusion and identified reproduction traits are essential targets of selection [9, 27, 28].

This assumption is supported by our detection of three consensus ROHs in all ten horses which harbor only one annotated gene, the *KITLG*, also known as *Mast Cell Growth Factor, Stem Cell Factor or steel factor* [59, 60]. Scans for signatures of diversifying selection in pigs proposed the *KITLG* locus to be a breed-specific signature in the Berkshire [61]. Due to its complex functional capacity, *KITLG* has fundamental impact on various essential processes affecting melanogenesis, haematopoiesis and gametogenesis [59, 62, 63]. Mutations in *KITLG* and its receptor *KIT* were shown to affect multiple cell formation stages parallelly during embryonic development and in fully-grown mice [63, 64]. The Steel Panda mutation at *KITLG* locus resulted in anemic black-eyed mice of white color with pigmented ears and scrotum and caused sterility in females. In human, a significant association for male infertility could be detected in *KITLG* affecting sperm count in patients [65]. In horses, the receptor of *KITLG* (*KIT)* was suggested to encode the dominant white (W) locus and to initiate severe disorders in haematopoietic system which might be responsible for the lethal consequences of homozygous W/W-genotype [66]. It was proposed that the dominant white phenotype is restricted in some breed registries due to the lethal effect of the homozygous dominant white mutation and also due to the risk of greater susceptibility to skin diseases. Therefore, we assume that the number of negative effects of *KITLG* mutations particularly affecting traits of reproduction and development have led to a strong positive selection of this region in horses that resulted in long ROHs.

The results of our study suggest that despite significant differences in-between breed and non-breed horses with regard to functional traits, all horse populations show strong signatures of selection in the region of genes affecting traits of reproduction.

## Methods

### Ethics statement

All animal work has been conducted according to the national and international guidelines for animal welfare. The EDTA-blood sampling was approved by the Institutional Animal Care and Use Committee (IACUC), the Lower Saxony state veterinary office at the Niedersächsisches Landesamt für Verbraucherschutz und Lebensmittelsicherheit, Oldenburg, Germany (registration number 11A 160/7221.3-2.1-015/11, 8.84-02.05.20.12.066).

### Samples and sequencing

Sequencing analysis was based on data from two Sorraia, one Dülmen Horse, one Arabian, one Saxon-Thuringian Heavy Warmblood, one Thoroughbred and four Hanoverian. Among these horses, six whole-genome sequences from two Hanoverian (SRX389480/SRX389477), one Arabian (SRX389472), one Sorraia (SRX389475), one Dülmen Horse (SRX384479) and one Thoroughbred (SRR1055837) were obtained from the Sequence Read Archive (NCBI). The remaining samples of a Sorraia mare, a Saxon-Thuringian Heavy Warmblood and two Hanoverian stallions were prepared for whole-genome sequencing. DNA was extracted from white blood cells derived from EDTA-blood sampling using Invisorb Spin Blood Mini kit according to the manufacturers’ protocol (Stratec Biomedical, Birkenfeld, Germany). Paired-end libraries of the two Hanoverian were prepared using the Illumina DNA sample preparation kit (Illumina, San Diego, CA). DNA-samples were sheared on the Covaris (Covaris, Woburn, Massachusetts) and purified with Agencourt AMPure XP beads (Beckman Coulter, Krefeld, Germany). The remaining two samples (Sorraia and Saxon-Thuringian Heavy Warmblood) were prepared using the Illumina Nextera DNA Sample Prep Kit according to the manufacturers’ protocol and purified with Agencourt AMPure XP beads as well. The whole genome of both Hanoverian was sequenced using an Illumina HiSeq2000 (Illumina) in paired-end mode (2 × 101 bp reads), whereas the Sorraia and Saxon-Thuringian Heavy Warmblood were run on an Illumina MiSeq with v2 Reagent Kits (2 x 250 bp reads) four times paired-end on a single lane flowcell to reach an adequate coverage for whole genome sequencing.

Quality control of FASTQ-files was done using fastqc 0.11.3 [67]. Reads were mapped to the reference genome EquCab2.70 using BWA 0.7.12 [68] and converted into binary format using SAMtools 1.2 [69]. PCR duplicated were marked using Picard tools (http://picard.sourceforge.net, version 1.130). Local realignment around INDELs, quality score recalibration and SNP calling was performed using GATK [70]. In order to get reliable data for variant detection we removed variants with a read depth <2 and >1000 and quality values <20 (qual). Variant annotation and effect prediction was done using SNPEff version 4.1 B (2015-02-13) [71]. The VCF file was adapted to PLINK 1.07 format using SAS/Genetics 9.4 (Statistical Analysis System, Cary, NC) and VCFtools 0.1.12b [72].

The data file for all ten horses re-sequenced is available at www.animalgenome.org (10horses.recode.vcf.gz). Raw data can be downloaded at the NCBI Sequence Read Archive (http://www.ncbi.nlm.nih.gov/sra), BioProject ID PRJNA291776 (Submission ID: SUB1048258).

### Runs of homozygosity

ROHs were detected using a trimmed dataset of 11,325,777 SNPs with a minimum read depth of 3, a maximum read depth of 60 and a minimum mean read depth of 12 for all ten samples. The X chromosome was omitted for this analysis. We defined ROHs as homozygous regions in sliding windows of 50 SNPs in a first run and 500 SNPs in a second approach using PLINK, version 1.07 (http://pngu.mgh.harvard.edu/purcell/plink/, [73]). Homozygous genotypes of >40 kb as well as >400 kb were defined as ROHs. The minimum distance of SNPs was estimated 0.8. This distance estimation was determined dividing the size of the genome covered with SNPs by the number of SNPs. No more than three SNPs with missing genotypes and three heterozygous SNPs were allowed in each window. The detected ROHs were categorized into small, medium and large ROHs and filtered for individual ROH regions for specific horses or breeds using SAS/Genetics, version 9.4. Private ROHs were determined by filtering out homozygous variants in ROHs in the horse of interest which could not be found in ROHs of other horses. Thus whole individual ROHs or individual parts of ROHs were detected as private ROHs for specific horses as well as for breeds or non-breeds. Consensus ROH regions were derived from intersections of homozygous variants in all ten horses. Furthermore inbreeding coefficients (F_ROH_) were estimated for each horse dividing the size of ROHs in bp by the length of the genome (2,242,879,462 bp) covered with SNPs.

In addition to that, theta estimations and neutrality test statistics Tajima’s D, Fu&Li F’s, Fu&Li’s D, Fay’s H, Zeng’s E were obtained using ANGSD version 0.902 [74]. Analyses were performed for all detected private ROHs in breed, non-breed and Hanoverian horses and for the consensus ROHs as well. Run parameters were adjusted to control for sequencing errors using a minimum quality value of 20 (−minQ 20) and filtering for a read depth of 3 to 60 (−geno_minDepth 3, −geno_maxDepth 60). Sliding windows of 40 kb as well as 400 kb were chosen for analysis.

### Sequence error detection by SNP50 BeadChip

In addition to whole-genome sequencing, two horses (Hanoverian) of a previous study [57] and three horses (two Hanoverian and one Arabian) of the current study were genotyped on the Illumina SNP50 BeadChip. Sequence errors were estimated in comparison with BeadChip data identifying heterozygous SNPs in BeadChip data which were homozygous in NGS data as false-negative and homozygous SNPs in BeadChip data which were heterozygous in NGS data as false-positive. For a more robust estimation of average false-positive error rates, long ROHs >1 Mb in sliding windows of 20 SNPs and a minimum distance of 50 were detected in BeadChip data using PLINK. No heterozygous SNPs and two missing called were admitted. These long ROH were assumed to hold error free homozygous genotypes and therefore ensure more precise error estimation in comparison with NGS-SNPs. The false-positive error rates were taken into account in the ROH detection admitting three heterozygous SNPs in each sliding window.

### Functional annotation

Gene lists of horse specific ROH regions were obtained using SAS/Genetics for filtering PLINK summary files and Galaxy intersection tool (https://usegalaxy.org/) [75–77] for gene allocation to genomic regions. The chromosomal positions of ROHs were aligned with the refseq gene table from UCSC (Ensembl genes) in order to obtain all genes located in ROHs. To improve functional analysis, we converted these gene lists to human orthologous genes using g:Profiler [78, 79]. PANTHER gene list analysis [80] was performed for functional classification of biological processes affected by genes in private ROH regions. In addition to these horse specific evaluations, further analyses for consensus ROHs in all ten horses and shared private ROHs in breed horses (Hanoverian, Arabian, Saxon-Thuringian Heavy Warmblood and Thoroughbred), non-breed horses (Dülmen Horse, Sorraia) and in the Hanoverian were performed. Gene names and its human orthologues were obtained using the Galaxy intersect function and g:Profiler as well. Genetic relations in-between genes were obtained using GeneMANIA [81].

### Functional variant detection

Functional variants with high or moderate effects were evaluated using SAS/Genetics for filtering SNPEff predictions categorized into high, moderate and low variant impacts. We determined the distribution of genotypes in relation to breed or non-breed groups and detected SIFT [82] prediction scores for functional effects using the Variant Effect Predictor [83].

## Additional files

Additional file 1:
**Summary of mapping metrics, sequence coverage and number of detected variants in ten horses.** (DOCX 174 kb) Additional file 2:
**Inbreeding coefficients (F**_**ROH**_ **) based on runs of homozygosity (ROH).** F_ROH_ was estimated dividing the total length of ROHs by the length of the genome covered by SNPs (2,242,879,462 bp). (DOCX 15 kb) Additional file 3:
**Functional annotations in private runs of homozygosity (ROH) of 500-SNP windows.** PANTHER gene list analysis (http://www.pantherdb.org/) was performed for genes in private ROH regions which could be exclusively found in one specific horse. The percent of gene hits against total number of process hits involved in specific biological processes are shown. (DOCX 18 kb) Additional file 4:
**Shared runs of homozygosity (ROHs) in non-breed horses.** The consensus private ROH regions and genes of the Dülmen Horse and two Sorraia in 50-SNP and 500-SNP windows are shown. The number of SNPs and size of shared ROH indicate the overlap of homozygous variants. (DOCX 78 kb) Additional file 5:
**Shared runs of homozygosity (ROHs) in breed horses.** The consensus private ROH regions and genes of Hanoverian, Arabian, Saxon-Thuringian Heavy Warmblood and Thoroughbred horses in 50-SNP windows are shown. The number of SNPs and size of shared ROH indicate the overlap of homozygous variants. (DOCX 14 kb) Additional file 6:
**Theta estimations and neutrality test statistics for consensus and private ROHs.** All private ROHs detected in the groups non-breed, breed and Hanoverian as well as in the region of *KITLG* were analyzed for Tajima’s D, Fu&Li F’s, Fu&Li’s D, Fay’s H, Zeng’s E using the software ANGSD (http://popgen.dk/angsd). (XLSX 18 kb) Additional file 7:
**Mutations with high or moderate effects in ROHs (50-SNP windows) of non-breed horses.** The ROH position and size (EquCab2.70), the position of SNPs, their mutant allele, potential impact and type are shown. Impact estimations are derived from SNPEff predictions. (DOCX 65 kb) Additional file 8:
**Mutations with high or moderate effects in ROHs (500-SNP windows) of non-breed horses.** The ROH position and size (EquCab2.70), the position of SNPs, their mutant allele, potential impact and type are shown. Impact estimations are derived from SNPEff predictions. (DOCX 16 kb) Additional file 9:
**Mutations with high or moderate effects in ROHs (50-SNP windows) of the four Hanoverians.** The ROH position and size (EquCab2.70), the position of SNPs, their mutant allele, potential impact and type are shown. Impact estimations are derived from SNPEff predictions. (DOCX 15 kb)

## Abbreviations

ROH: Runs of homozygosity
SNP: Single nucleotide polymorphism
NGS: Next generation sequencing
*MSTN*: *Myostatin* gene
*PLAG1*: *Pleiomorphic adenoma gene 1*
*EDNRB*: *Endothelin receptor type B*, *DYX1C1, dyslexia susceptibility 1 candidate 1*
*PPP1R14C*: *Protein phosphatase 1, regulatory (inhibitor) subunit 14C*
*CFAP61/C20orf26*: *Cilia and flagella associated protein 61*
*CSAD*: *Cysteine sulfinic acid decarboxylase*
*TBC1D30*: *TBC1 domain family, member 30*
*ALX4*: *ALX homeobox 4*
*KITLG*: *KIT Ligand*
