# Supplementary material for: Runs of homozygosity reveal signatures of positive selection for reproduction traits in breed and non-breed horses
Source: BMC Genomics. 2015 Oct 9;16:764. doi: 10.1186/s12864-015-1977-3 (PMC4600213; doi:10.1186/s12864-015-1977-3)

Additional file 1. Summary of mapping metrics, sequence coverage and number of detected variants in ten horses.

|  | Horse 1  Dülmen  Horse | Horse 2  Sorraia | Horse 3  Sorraia | Horse 4  Hanoverian | Horse 5  Hanoverian | Horse 6  Hanoverian | Horse 7  Hanoverian | Horse 8  Saxon-Thuringian Heavy Warmblood | Horse 9  Arabian | Horse 10  Thoroughbred  (SRR1055837) |
| --- | --- | --- | --- | --- | --- | --- | --- | --- | --- | --- |
| Platform | Illumina HiSeq 2000 | Illumina HiSeq 2000 | Illumina MiSeq | Illumina HiSeq 2000 | Illumina HiSeq 2000 | Illumina HiSeq 2000 | Illumina HiSeq 2000 | Illumina MiSeq | Illumina HiSeq 2000 | Illumina HiSeq 2000 |
| Number of lanes | 1 | 1 | 4 | 2 | 1 | 1 | 1 | 4 | 1 | 1 |
| **Yield summary** |  |  |  |  |  |  |  |  |  |  |
| raw total sequences | 401962333 | 357784225 | 144547516 | 719032676 | 317323076 | 303463999 | 319288414 | 162964754 | 386657685 | 444034325 |
| average read length | 99 | 99 | 240 | 99 | 99 | 100 | 100 | 243 | 99 | 99 |
| maximum read length | 100 | 100 | 251 | 100 | 100 | 101 | 101 | 251 | 100 | 100 |
| **Mapping Summary** |  |  |  |  |  |  |  |  |  |  |
| reads mapped | 383605550 | 335328011 | 142906254 | 679776083 | 295734339 | 293319305 | 309136383 | 161037793 | 367191888 | 425310825 |
| reads unmapped | 18356783 | 22456214 | 1641262 | 39256593 | 21588737 | 10144694 | 10152031 | 1926961 | 19465797 | 18723500 |
| bases mapped | 38299333881 | 33465153944 | 34295699208 | 67881552084 | 29531691639 | 29585597442 | 31180915452 | 39150275334 | 36660298237 | 42487260348 |
| **Coverage** |  |  |  |  |  |  |  |  |  |  |
| Mean coverage | 19.90 | 17.55 | 17.34 | 35.18 | 15.85 | 15.66 | 16.46 | 20.06 | 19.14 | 5.92 |
| **Variant detection** |  |  |  |  |  |  |  |  |  |  |
| Number of SNPs | 7036126 | 6746445 | 6835500 | 6923412 | 6734179 | 6700451 | 6680535 | 7147081 | 6856413 | 3865613 |
| Number of INDELs | 958085 | 927229 | 970891 | 959022 | 919587 | 905461 | 905257 | 992338 | 938810 | 698724 |


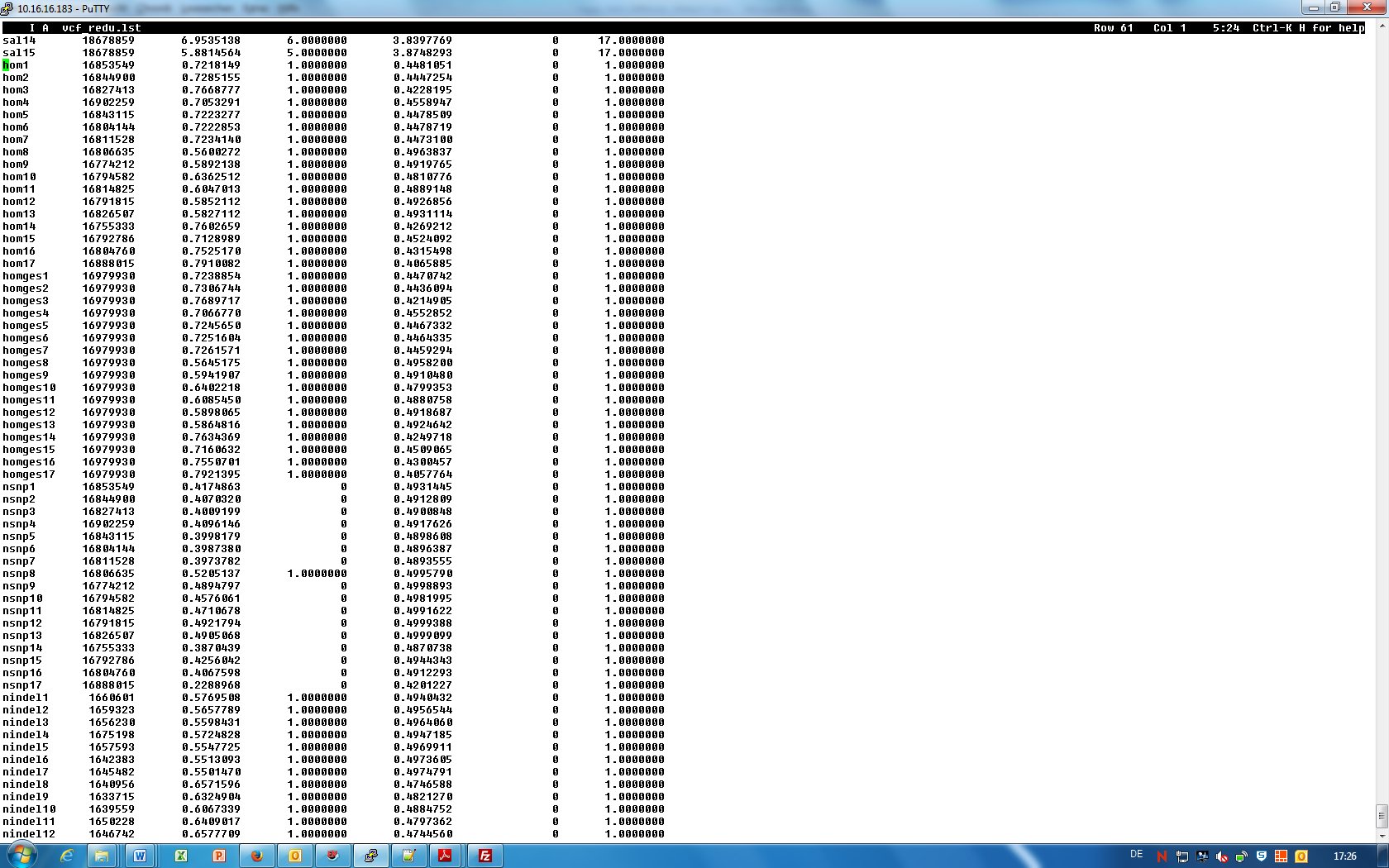

Supplement: Additional file 1: — Summary of mapping metrics, sequence coverage and number of detected variants in ten horses. (DOCX 174 kb) [file 12864_2015_1977_MOESM1_ESM.docx]
