# Supplementary material for: Runs of homozygosity reveal signatures of positive selection for reproduction traits in breed and non-breed horses
Source: BMC Genomics. 2015 Oct 9;16:764. doi: 10.1186/s12864-015-1977-3 (PMC4600213; doi:10.1186/s12864-015-1977-3)
Supplement: Additional file 2: — Inbreeding coefficients (F ROH ) based on runs of homozygosity (ROH). FROH was estimated dividing the total length of ROHs by the length of the genome covered by SNPs (2,242,879,462 bp). (DOCX 15 kb) [file 12864_2015_1977_MOESM2_ESM.docx]

Additional file 2. Inbreeding coefficients (F_ROH_) based on runs of homozygosity (ROH). F_ROH_ was estimated dividing the total length of ROHs by the length of the genome covered by SNPs (2242879462 bp).

| Horse | Breed | Total length of ROHs (bp) in sliding windows of at least 50 SNPs | F_ROH50_ | Total length of ROHs (bp) in sliding windows of at least 500 SNPs | F_ROH500_ |
| --- | --- | --- | --- | --- | --- |
| Horse 1 | Dülmen Horse | 416558082 | 0.18572 | 123996902 | 0.05528 |
| Horse 2 | Sorraia | 867014755 | 0.38656 | 351410803 | 0.15668 |
| Horse 3 | Sorraia | 730247430 | 0.32558 | 272039173 | 0.12129 |
| Horse 4 | Hanoverian | 408729764 | 0.18223 | 87642889 | 0.03908 |
| Horse 5 | Hanoverian | 490390260 | 0.21864 | 130547202 | 0.05821 |
| Horse 6 | Hanoverian | 460635582 | 0.20538 | 110871138 | 0.04943 |
| Horse 7 | Hanoverian | 456348323 | 0.20347 | 106090548 | 0.04730 |
| Horse 8 | Saxon-Thuringian Heavy Warmblood | 476060417 | 0.21225 | 127748644 | 0.05696 |
| Horse 9 | Arabian | 565573536 | 0.25216 | 159400131 | 0.07107 |
| Horse 10 (SRR1055837) | Thoroughbred | 953192320 | 0.42499 | 407085456 | 0.18150 |
