# Supplementary material for: Runs of homozygosity reveal signatures of positive selection for reproduction traits in breed and non-breed horses
Source: BMC Genomics. 2015 Oct 9;16:764. doi: 10.1186/s12864-015-1977-3 (PMC4600213; doi:10.1186/s12864-015-1977-3)
Supplement: Additional file 3: — Functional annotations in private runs of homozygosity (ROH) of 500-SNP windows. PANTHER gene list analysis (http://www.pantherdb.org/) was performed for genes in private ROH regions which could be exclusively found in one specific horse. The percent of gene hits against total number of process hits involved in specific biological processes are shown. (DOCX 18 kb) [file 12864_2015_1977_MOESM3_ESM.docx]

Additional file 3. Functional annotations in private runs of homozygosity (ROH) of 500-SNP windows. PANTHER gene list analysis (http://www.pantherdb.org/) was performed for genes in private ROH regions which could be exclusively found in one specific horse. The percent of gene hits against total number of process hits involved in specific biological processes are shown.

| PANTHER gene ontology terms | Horse 1  Dülmen  Horse (%) | Horse 2  Sorraia (%) | Horse 3  Sorraia (%) | Horse 4  Hanoverian (%) | Horse 5  Hanoverian (%) | Horse 6  Hanoverian (%) | Horse 7  Hanoverian (%) | Horse 8  Saxon-Thuringian Heavy Warmblood (%) | Horse Arabian (%) | Horse 10  Thoroughbred (SRR1055837) (%) |
| --- | --- | --- | --- | --- | --- | --- | --- | --- | --- | --- |
| cellular component organization or biogenesis (GO:0071840) | 5.0 | 3.4 | 2.2 | 6.0 | 3.8 | 5.4 | 3.8 | 5.9 | 4.5 | 3.3 |
| cellular process (GO:0009987) | 17.5 | 20.3 | 21.0 | 20.5 | 18.5 | 22.7 | 23.5 | 20.2 | 18.7 | 18.7 |
| localization (GO:0051179) | 10.7 | 9.1 | 8.1 | 12.5 | 9.7 | 9.4 | 8.0 | 8.9 | 11.4 | 7.7 |
| apoptotic process (GO:0006915) | 1.2 | 1.7 | 2.6 | 2.0 | 2.3 | 1.4 | 0.8 | 1.7 | 1.9 | 2.5 |
| reproduction (GO:0000003) | 1.2 | 1.4 | 2.1 | 0.5 | 1.5 | 0.6 | 0.8 | 1.7 | 1.4 | 2.0 |
| biological regulation (GO:0065007) | 10.2 | 8.9 | 10.0 | 10.5 | 8.7 | 9.7 | 10.1 | 8.4 | 8.4 | 9.9 |

Additional file 3 continued.

| PANTHER gene ontology terms | Horse 1  Dülmen  Horse (%) | Horse 2  Sorraia (%) | Horse 3  Sorraia (%) | Horse 4  Hanoverian (%) | Horse 5  Hanoverian (%) | Horse 6  Hanoverian (%) | Horse 7  Hanoverian (%) | Horse 8  Saxon-Thuringian Heavy Warmblood (%) | Horse Arabian (%) | Horse 10  Thoroughbred (SRR1055837) (%) |
| --- | --- | --- | --- | --- | --- | --- | --- | --- | --- | --- |
| response to stimulus (GO:0050896) | 3.0 | 6.4 | 6.3 | 0.5 | 4.4 | 4.5 | 1.7 | 6.7 | 3.9 | 5.0 |
| developmental process (GO:0032502) | 9.0 | 7.2 | 7.7 | 11.5 | 9.5 | 10.2 | 10.1 | 9.9 | 10.3 | 9.7 |
| multicellular organismal process (GO:0032501) | 7.7 | 5.8 | 4.5 | 4.0 | 5.9 | 5.1 | 8.0 | 6.4 | 5.0 | 7.1 |
| locomotion (GO:0040011) | 0.0 | 0.1 | 0.0 | 0.0 | 0.0 | 0.0 | 0.0 | 0.2 | 0.0 | 0.0 |
| biological adhesion (GO:0022610) | 2.2 | 3.8 | 4.0 | 3.0 | 2.8 | 4.0 | 3.8 | 4.4 | 2.8 | 3.3 |
| metabolic process (GO:0008152) | 28.2 | 26.3 | 24.3 | 27.0 | 27.9 | 22.7 | 26.5 | 19.3 | 27.3 | 25.2 |
| immune system process (GO:0002376) | 4.0 | 5.7 | 7.2 | 2.0 | 4.9 | 4.3 | 2.9 | 6.2 | 4.5 | 5.5 |
