# Supplementary material for: Runs of homozygosity reveal signatures of positive selection for reproduction traits in breed and non-breed horses
Source: BMC Genomics. 2015 Oct 9;16:764. doi: 10.1186/s12864-015-1977-3 (PMC4600213; doi:10.1186/s12864-015-1977-3)
Supplement: Additional file 4: — Shared runs of homozygosity (ROHs) in non-breed horses. The consensus private ROH regions and genes of the Dülmen Horse and two Sorraia in 50-SNP and 500-SNP windows are shown. The number of SNPs and size of shared ROH indicate the overlap of homozygous variants. (DOCX 78 kb) [file 12864_2015_1977_MOESM4_ESM.docx]

Additional file 4. Shared runs of homozygosity (ROHs) in non-breed horses. The consensus private ROH regions and genes of the Dülmen Horse and two Sorraia in 50-SNP and 500-SNP windows are shown. The number of SNPs and size of shared ROH indicate the overlap of homozygous variants.

| ECA | Position | Number of SNPs in shared parts of ROH regions | Size of shared ROH (bp) | Gene ID | Human ortholog | Gene name/ function |
| --- | --- | --- | --- | --- | --- | --- |
| **50-SNP window** |  |  |  |  |  |  |
| 1 | 8453180-8463384 | 15 | 10205 | ENSECAT00000010323 | *LHPP* | phospholysine phosphor-histidine inorganic pyrophosphate phosphatase |
| 1 | 119028193-119028193 | 1 | 1 | - | *-* | - |
| 1 | 129290715-129374751 | 251 | 84037 | ENSECAT00000023339, ENSECAT00000023447, ENSECAT00000023476 | *TLN2* | Talin 2 |
| 1 | 129886736-129933585 | 208 | 46850 | ENSECAT00000019241 | *VPS13C* | vacuolar protein sorting 13 homolog C (S. cerevisiae) |
| 1 | 129934428-130014163 | 396 | 79736 | ENSECAT00000019241 | *VPS13C* | vacuolar protein sorting 13 homolog C (S. cerevisiae) |
| 1 | 130054318-130129275 | 346 | 74958 | ENSECAT00000016401, ENSECAT00000029559 | *novel gene,*  *novel gene* |  |
| 1 | 138002506-138003882 | 7 | 1377 | ENSECAT00000014788 | *FAM214A* | family with sequence similarity 214, member A |
| 1 | 138467809-138467846 | 4 | 38 | - | *-* | - |

Additional file 4. continued

| ECA | Position | Number of SNPs in shared parts of ROH regions | Size of shared ROH (bp) | Gene ID | Human ortholog | Gene name/ function |
| --- | --- | --- | --- | --- | --- | --- |
| 1 | 177634055-177634055 | 1 | 1 | - | *-* | - |
| 2 | 79888517-80111836 | 541 | 223320 | ENSECAT00000018012 | *SFRP2* | secreted frizzled-related protein 2 |
| 2 | 85868575-85871648 | 14 | 3074 | - | *-* | - |
| 2 | 85872597-85926439 | 143 | 53843 | - | *-* | - |
| 3 | 54140166-54149254 | 19 | 9089 | ENSECAT00000009353 | *LIN54* | lin-54 DREAM MuvB core complex component |
| 3 | 54149532-54149532 | 1 | 1 | - | *-* | - |
| 3 | 54532191-54532191 | 1 | 1 | - | *-* | - |
| 3 | 54911041-54911081 | 3 | 41 | - | *-* | - |
| 3 | 58133505-58337269 | 871 | 203765 | ENSECAT00000008119 | *FRAS1* | fraser extracellular matrix complex subunit 1 |
| 3 | 69577573-69646630 | 366 | 69058 | - | *-* | - |
| 3 | 80124927-80137896 | 52 | 12970 | - | *-* | - |
| 3 | 80236787-80236787 | 1 | 1 | - | *-* | - |
| 3 | 92257746-92347841 | 559 | 90096 | - | *-* | - |
| 4 | 2006511-2057578 | 271 | 51068 | - | *-* | - |
| 4 | 2852363-2852369 | 2 | 7 | ENSECAT00000009031 | *MAGI2* | membrane associated guanylate kinase, WW and PDZ domain containing 2 |
|  |  |  |  |  |  |  |

Additional file 4. continued

| ECA | Position | Number of SNPs in shared parts of ROH regions | Size of shared ROH (bp) | Gene ID | Human ortholog | Gene name/ function |
| --- | --- | --- | --- | --- | --- | --- |
| 4 | 39707012-39707021 | 2 | 10 | - | *-* | - |
| 4 | 39776894-39832480 | 438 | 55587 | ENSECAT00000001132 | *novel gene* |  |
| 4 | 39833171-39842378 | 64 | 9208 | ENSECAT00000013801 | *novel gene* |  |
| 4 | 39947000-39990749 | 129 | 43750 | - | *-* | - |
| 4 | 56883978-56884187 | 5 | 210 | - | *-* | - |
| 5 | 5352975-5380533 | 143 | 27559 | - | *-* | - |
| 5 | 5381094-5402484 | 101 | 21391 | ENSECAT00000018667 | *novel gene* |  |
| 5 | 6081735-6118055 | 154 | 36321 | ENSECAT00000021448 | *SELL* | selectin L |
| 5 | 8009556-8060343 | 189 | 50788 | ENSECAT00000029159, ENSECAT00000010913, ENSECAT00000010907 | *DNM3,*  *DNM3,*  *DNM3* | dynamin 3 |
| 5 | 8591503-8623292 | 137 | 31790 | ENSECAT00000029694 | *novel gene* |  |
| 5 | 8623428-8623594 | 3 | 167 | - | *-* | - |
| 5 | 8629238-8703261 | 359 | 74024 | - | *-* | - |
| 5 | 24256664-24256820 | 3 | 157 | - | *-* | - |
| 5 | 58362207-58424096 | 36 | 61890 | ENSECAT00000018749, ENSECAT00000019106, ENSECAT00000020568 | *CSF1,*  *EPS8L3,*  *GSTM3* | colony stimulating factor 1 (macrophage),  EPS8-like 3,  glutathione S-transferase mu 3 (brain) |
| 5 | 62456604-62502452 | 325 | 45849 | - | *-* | - |

Additional file 4. continued

| ECA | Position | Number of SNPs in shared parts of ROH regions | Size of shared ROH (bp) | Gene ID | Human ortholog | Gene name/ function |
| --- | --- | --- | --- | --- | --- | --- |
| 5 | 76184204-76188295 | 30 | 4092 | - | *-* | - |
| 5 | 76335123-76338010 | 20 | 2888 | - | *-* | - |
| 5 | 88969506-88969717 | 2 | 212 | - | *-* | - |
| 6 | 46798367-46841287 | 143 | 42921 | - | *-* | - |
| 6 | 72134510-72134774 | 3 | 265 | - | *-* | - |
| 7 | 9929774-9930127 | 4 | 354 | ENSECAT00000001108 | *CNTN5* | contactin 5 |
| 7 | 30059891-30062856 | 15 | 2966 | ENSECAT00000019982 | *UBASH3B* | ubiquitin associated and SH3 domain containing B |
| 7 | 30164720-30222327 | 121 | 57608 | ENSECAT00000011227, ENSECAT00000013340 | *C11ORF63,*  *BSX* | chromosome 11 open reading frame 63, brain-specific homeobox |
| 7 | 32594706-32595616 | 12 | 911 |  |  |  |
| 7 | 85593736-85620317 | 121 | 26582 | ENSECAT00000017896, ENSECAT00000017959 | *NUCB2,*  *NUCB2* | nucleobindin 2 |
| 9 | 19746074-19795257 | 231 | 49184 | - | *-* | - |
| 9 | 20320347-20360083 | 154 | 39737 | ENSECAT00000007698, ENSECAT00000008930 | *NUCB2,*  *novel gene* | nucleobindin 2 |
| 9 | 65689063-65773640 | 214 | 84578 | ENSECAT00000020108, ENSECAT00000022212, ENSECAT00000025099 | *FAM83A,*  *C8ORF76,*  *ZHX1* | family with sequence similarity 83, member A, chromosome 8 open reading frame 76,  zinc fingers and homeoboxes 1 |

Additional file 4. continued

| ECA | Position | Number of SNPs in shared parts of ROH regions | Size of shared ROH (bp) | Gene ID | Human ortholog | Gene name/ function |
| --- | --- | --- | --- | --- | --- | --- |
| 10 | 14606245-14628891 | 16 | 22647 | ENSECAT00000014330, ENSECAT00000014776 | *ZNF428,*  *CADM4* | zinc finger protein 428,  cell adhesion molecule 4 |
| 10 | 14630027-14653098 | 74 | 23072 | ENSECAT00000023704 | *PLAUR* | plasminogen activator, urokinase receptor |
| 10 | 14737181-14737444 | 7 | 264 | - | *-* | - |
| 10 | 15738781-15782429 | 58 | 43649 | ENSECAT00000028998, ENSECAT00000020225, ENSECAT00000024295 | *DACT3, TRAPPC6A,*  *novel gene* | dishevelled-binding antagonist of beta-catenin 3,  trafficking protein particle complex 6A |
| 10 | 15782483-15858844 | 140 | 76362 | ENSECAT00000028998, ENSECAT00000000882, ENSECAT00000001234, ENSECAT00000024682, ENSECAT00000025644 | *DACT3,*  *MARK4,*  *MARK4,*  *novel gene, EXOC3L2* | dishevelled-binding antagonist of beta-catenin 3, MAP/microtubule affinity-regulating kinase 4,  exocyst complex component 3-like 2 |
| 10 | 17034967-17104150 | 148 | 69184 | ENSECAT00000018888 | *ARHGAP35* | Rho GTPase activating protein 35 |
|  |  |  |  |  |  |  |

Additional file 4. continued

| ECA | Position | Number of SNPs in shared parts of ROH regions | Size of shared ROH (bp) | Gene ID | Human ortholog | Gene name/ function |
| --- | --- | --- | --- | --- | --- | --- |
| 10 | 19028284-19069077 | 66 | 40794 | ENSECAT00000004206, ENSECAT00000021257, ENSECAT00000023252 | *PPFIA3,*  *HRC,*  *TRPM4* | protein tyrosine phosphatase, receptor type, F polypeptide (PTPRF), interacting protein (liprin), alpha 3,  histidine rich calcium binding protein, transient receptor potential cation channel, subfamily M, member 4 |
| 10 | 20896796-20961513 | 327 | 64718 | ENSECAT00000026015, ENSECAT00000025272, ENSECAT00000025953 | *novel gene,*  *novel gene, SIGLECL1* | SIGLEC family like 1 |
| 10 | 21033978-21033978 | 1 | 1 | - | *-* | - |
| 10 | 21089949-21100824 | 41 | 10876 | - | *-* | - |
| 10 | 22103520-22103520 | 1 | 1 | - | *-* | - |
| 10 | 22165158-22227140 | 388 | 61983 | ENSECAT00000000805, ENSECAT00000024671, ENSECAT00000000844, ENSECAT00000000869, ENSECAT00000000936 | *novel gene,*  *novel gene,*  *novel gene,*  *novel gene,*  *novel gene* |  |
| 10 | 22228721-22283734 | 302 | 55014 | ENSECAT00000024688, ENSECAT00000024706 | *ZNF610,*  *novel gene* | zinc finger protein 610 |

Additional file 4. continued

| ECA | Position | Number of SNPs in shared parts of ROH regions | Size of shared ROH (bp) | Gene ID | Human ortholog | Gene name/ function |
| --- | --- | --- | --- | --- | --- | --- |
| 10 | 32049398-32133977 | 442 | 84580 | ENSECAT00000007041 | *MEI4* | meiosis-specific 4 homolog (S. cerevisiae) |
| 10 | 32251893-32280105 | 155 | 28213 | - | *-* | - |
| 10 | 32393657-32393657 | 1 | 1 | - | *-* | - |
| 10 | 32936208-32969407 | 106 | 33200 | - | *-* | - |
| 10 | 32969951-33026593 | 219 | 56643 | ENSECAT00000008363 | *novel gene* |  |
| 10 | 33027898-33067713 | 119 | 39816 | ENSECAT00000004153 | *novel gene* |  |
| 10 | 33069658-33070748 | 5 | 1091 | ENSECAT00000008375 | *novel gene* |  |
| 10 | 33071963-33071963 | 1 | 1 | - | *-* | - |
| 10 | 33115495-33117134 | 9 | 1640 | - | *-* | - |
| 10 | 33137125-33461831 | 1344 | 324707 | ENSECAT00000009409, ENSECAT00000011576, ENSECAT00000009390 | *IRAK1BP1,*  *PHIP,*  *novel gene* | interleukin-1 receptor-associated kinase 1 binding protein 1, pleckstrin homology domain interacting protein |
| 10 | 33461856-33489347 | 114 | 27492 | ENSECAT00000021226 | *HMGN3* | high mobility group nucleosomal binding domain 3 |
| 10 | 34152280-34237984 | 276 | 85705 | ENSECAT00000024536, ENSECAT00000025743 | *ELOVL4,*  *TTK* | ELOVL fatty acid elongase 4, TTK protein kinase |
| 10 | 34281410-34281410 | 1 | 1 | - | *-* | - |

Additional file 4. continued

| ECA | Position | Number of SNPs in shared parts of ROH regions | Size of shared ROH (bp) | Gene ID | Human ortholog | Gene name/ function |
| --- | --- | --- | --- | --- | --- | --- |
| 10 | 34292539-34338940 | 95 | 46402 | ENSECAT00000010232 | *BCKDHB* | branched chain keto acid dehydrogenase E1, beta polypeptide |
| 10 | 34339101-34485897 | 448 | 146797 | ENSECAT00000010232 | *BCKDHB* | branched chain keto acid dehydrogenase E1, beta polypeptide |
| 10 | 35320857-35340212 | 65 | 19356 | - | *-* | - |
| 10 | 38332023-38362031 | 149 | 30009 | - | *-* | - |
| 10 | 38434346-38487176 | 311 | 52831 | - | *-* | - |
| 10 | 39065010-39108510 | 218 | 43501 | ENSECAT00000016349 | *novel gene* |  |
| 10 | 39108791-39134999 | 227 | 26209 | - | *-* | - |
| 10 | 39168419-39185110 | 122 | 16692 | - | *-* | - |
| 10 | 39185413-39272324 | 505 | 86912 | ENSECAT00000016363 | *novel gene* |  |
| 10 | 58389003-58389144 | 2 | 142 | - | *-* | - |
| 11 | 8384586-8421188 | 86 | 36603 | ENSECAT00000005181, ENSECAT00000008658 | *FAM104A,*  *COG1* | family with sequence similarity 104, member A,  component of oligomeric golgi complex 1 |
| 11 | 41904947-41945734 | 79 | 40788 | ENSECAT00000026831, ENSECAT00000026843 | *NOS2,*  *NOS2* | nitric oxide synthase 2, inducible |
| 12 | 20482-20496 | 2 | 15 | - | *-* | - |

Additional file 4. continued

| ECA | Position | Number of SNPs in shared parts of ROH regions | Size of shared ROH (bp) | Gene ID | Human ortholog | Gene name/ function |
| --- | --- | --- | --- | --- | --- | --- |
| 12 | 17555415-17556067 | 4 | 653 | ENSECAT00000008594 | *SLC43A1* | solute carrier family 43 (amino acid system L transporter), member 1 |
| 13 | 18036672-18036940 | 7 | 269 | - | *-* | - |
| 13 | 23251693-23292865 | 140 | 41173 | ENSECAT00000016958 | *CACNG3* | calcium channel, voltage-dependent, gamma subunit 3 |
| 13 | 26219276-26219291 | 4 | 16 | ENSECAT00000024567 | *novel gene* |  |
| 13 | 32790136-32887855 | 341 | 97720 | ENSECAT00000005954, ENSECAT00000014164, ENSECAT00000003938 | *ZC3H7A,*  *TXNDC11,*  *SNN* | zinc finger CCCH-type containing 7A,  thioredoxin domain containing 11, stannin |
| 14 | 41060852-41076339 | 28 | 15488 | ENSECAT00000018129, ENSECAT00000019747 | *C5ORF24,*  *DDX46* | chromosome 5 open reading frame 24,  DEAD (Asp-Glu-Ala-Asp) box polypeptide 46 |
| 14 | 63779564-63791998 | 49 | 12435 | - | *-* | - |
| 14 | 76745952-76754403 | 39 | 8452 | - | *-* | - |
| 14 | 86901424-86901468 | 4 | 45 | - | *-* | - |
| 14 | 91321472-91321472 | 1 | 1 | - | *-* | - |
| 14 | 91460880-91460914 | 5 | 35 | - | *-* | - |
| 15 | 70989370-70989753 | 3 | 384 | ENSECAT00000009021 | *ADCY3* | adenylate cyclase 3 |

Additional file 4. continued

| ECA | Position | Number of SNPs in shared parts of ROH regions | Size of shared ROH (bp) | Gene ID | Human ortholog | Gene name/ function |
| --- | --- | --- | --- | --- | --- | --- |
| 16 | 66073871-66073895 | 2 | 25 | ENSECAT00000026211 | *EAF1* | ELL associated factor 1 |
| 18 | 2679677-2680375 | 14 | 699 | ENSECAT00000023316 | *UGGT1* | UDP-glucose glycoprotein glucosyltransferase 1 |
| 18 | 3004825-3004839 | 3 | 15 | ENSECAT00000001765 | *WDR33* | WD repeat domain 33 |
| 18 | 3066502-3066713 | 11 | 212 | ENSECAT00000001765 | *WDR33* | WD repeat domain 33 |
| 18 | 3857257-3933135 | 379 | 75879 | ENSECAT00000028761 | *novel gene* |  |
| 18 | 3990269-4001279 | 39 | 11011 | - | *-* | - |
| 18 | 4192658-4233607 | 79 | 40950 | - | *-* | - |
| 18 | 4707088-4718119 | 201 | 11032 | - | *-* | - |
| 18 | 4807529-4807577 | 5 | 49 | - | *-* | - |
| 18 | 4889976-4915104 | 169 | 25129 | ENSECAT00000001796 | *novel gene* |  |
| 18 | 5813011-5813109 | 2 | 99 | - | *-* | - |
| 18 | 6004226-6004226 | 1 | 1 | ENSECAT00000029118 | *CNTNAP5* | contactin associated protein-like 5 |
| 18 | 6098052-6098214 | 7 | 163 | ENSECAT00000029118, ENSECAT00000019453 | *CNTNAP5, CNTNAP5* | contactin associated protein-like 5 |
| 18 | 12952561-13007393 | 191 | 54833 | ENSECAT00000012467, ENSECAT00000012537 | *CCDC93,*  *CCDC93* | coiled-coil domain containing 93 |
| 18 | 15979464-15986617 | 49 | 7154 | - | *-* | - |
| 18 | 15986944-15991007 | 25 | 4064 | - | *-* | - |
| 18 | 16034100-16034722 | 7 | 623 | - | *-* | - |

Additional file 4. continued

| ECA | Position | Number of SNPs in shared parts of ROH regions | Size of shared ROH (bp) | Gene ID | Human ortholog | Gene name/ function |
| --- | --- | --- | --- | --- | --- | --- |
| 18 | 17703638-17712663 | 32 | 9026 | - | *-* | - |
| 20 | 46698001-46699360 | 8 | 1360 | - | *-* | - |
| 20 | 46797292-46797780 | 3 | 489 | - | *-* | - |
| 21 | 14424382-14428239 | 14 | 3858 | - | *-* | - |
| 21 | 17220183-17220349 | 8 | 167 | - | *-* | - |
| 21 | 48575054-48591376 | 79 | 16323 | ENSECAT00000028918, ENSECAT00000009423 | *CTNND2,*  *CTNND2* | catenin (cadherin-associated protein), delta |
| 21 | 49837032-49951766 | 374 | 114735 | ENSECAT00000005035, ENSECAT00000029372 | *TAS2R1,*  *SNORD123* | taste receptor, type 2, member, small nucleolar RNA, C/D box 123 |
| 21 | 50011315-50022412 | 53 | 11098 | - | *-* | - |
| 21 | 50023234-50119009 | 240 | 95776 | ENSECAT00000017338 | *SEMA5A* | sema domain, seven thrombo- spondin repeats (type 1 and type 1-like), transmembrane domain (TM) and short cytoplasmic domain, (semaphorin) 5A |
| 21 | 50270718-50330925 | 330 | 60208 | ENSECAT00000017338 | *SEMA5A* | sema domain, seven thrombo-spondin repeats (type 1 and type 1-like), transmembrane domain (TM) and short cytoplasmic domain, (semaphorin) 5A |

Additional file 4. continued

| ECA | Position | Number of SNPs in shared parts of ROH regions | Size of shared ROH (bp) | Gene ID | Human ortholog | Gene name/ function |
| --- | --- | --- | --- | --- | --- | --- |
| 21 | 50421106-50427010 | 56 | 5905 | - | *-* | - |
| 21 | 51081671-51106060 | 130 | 24390 | - | *-* | - |
| 22 | 14951698-14951726 | 3 | 29 | ENSECAT00000022663 | *PLCB1* | phospholipase C, beta 1 |
| 22 | 27255689-27267779 | 45 | 12091 | - | *-* | - |
| 22 | 33133065-33179342 | 131 | 46278 | - | *-* | - |
| 22 | 33262137-33304967 | 141 | 42831 | ENSECAT00000011825 | *TOX2* | TOX high mobility group box family member 2 |
| 22 | 33434602-33508238 | 196 | 73637 | ENSECAT00000017678, ENSECAT00000020011 | *JPH2,*  *OSER1* | junctophilin 2,  oxidative stress responsive serine-rich 1 |
| 22 | 33577807-33633125 | 134 | 55319 | ENSECAT00000025609, ENSECAT00000026400, ENSECAT00000026421 | *R3HDML,*  *HNF4A,*  *HNF4A* | R3H domain containing-like, hepatocyte nuclear factor 4, alpha |
| 22 | 33730758-33741334 | 66 | 10577 | - | *-* | - |
| 22 | 33741915-33848551 | 233 | 106637 | ENSECAT00000008811, ENSECAT00000007863 | *ADA,*  *PKIG* | adenosine deaminase,  protein kinase (cAMP-dependent, catalytic) inhibitor gamma |
| 22 | 34009075-34019069 | 70 | 9995 | - | *-* | - |

Additional file 4. continued

| ECA | Position | Number of SNPs in shared parts of ROH regions | Size of shared ROH (bp) | Gene ID | Human ortholog | Gene name/ function |
| --- | --- | --- | --- | --- | --- | --- |
| 22 | 34023768-34136641 | 328 | 112874 | ENSECAT00000024818, ENSECAT00000021794, ENSECAT00000021700 | *PABPC1L,*  *YWHAB,*  *TOMM34* | poly(A) binding protein, cytoplasmic 1-like,  tyrosine 3-monooxygenase/tryptophan 5-monooxygenase activation protein, beta,  translocase of outer mitochondrial membrane 34 |
| 22 | 34136970-34241197 | 601 | 104228 | ENSECAT00000023629, ENSECAT00000010717, ENSECAT00000014615 | *STK4,*  *KCNS1,*  *WFDC5* | serine/threonine kinase 4, potassium voltage-gated channel, modifier subfamily S, member 1,  WAP four-disulfide core domain 5 |
| 22 | 34480730-34515314 | 99 | 34585 | ENSECAT00000001962, ENSECAT00000001317 | *novel gene,*  *novel gene* |  |
| 22 | 34562233-34614304 | 203 | 52072 | ENSECAT00000023076, ENSECAT00000020282, ENSECAT00000020297, ENSECAT00000021020 | *WFDC8,*  *WFDC6,*  *WFDC6,*  *EPPIN-WFDC6* | WAP four-disulfide core domain 8,  WAP four-disulfide core domain 6,  EPPIN-WFDC6 readthrough |

Additional file 4. continued

| ECA | Position | Number of SNPs in shared parts of ROH regions | Size of shared ROH (bp) | Gene ID | Human ortholog | Gene name/ function |
| --- | --- | --- | --- | --- | --- | --- |
| 22 | 34664316-34722972 | 214 | 58657 | ENSECAT00000024363, ENSECAT00000024373 | *WFDC13,*  *SPINT4* | WAP four-disulfide core domain 13,  serine peptidase inhibitor, Kunitz type 4 |
| 22 | 34725011-34738028 | 58 | 13018 | - | *-* | - |
| 22 | 34768055-34931170 | 234 | 163116 | ENSECAT00000021279, ENSECAT00000025003, ENSECAT00000015457, ENSECAT00000021003, ENSECAT00000023028, ENSECAT00000023435, ENSECAT00000001514, ENSECAT00000001375, ENSECAT00000001682, ENSECAT00000001920, ENSECAT00000005323, ENSECAT00000020342, ENSECAT00000020385, ENSECAT00000025620 | *PLTP,*  *DNTTIP1,*  *UBE2C,*  *TNNC2,*  *SNX21,*  *ACOT8,*  *ZSWIM3,*  *ZSWIM3,*  *SPATA25,*  *NEURL2,*  *CTSA,*  *PCIF1,*  *PCIF1,*  *ZNF335* | phospholipid transfer protein, deoxynucleotidyltransferase, terminal, interacting protein 1, ubiquitin-conjugating enzyme E2C,  troponin C type 2 (fast),  sorting nexin family member 21,  acyl-CoA thioesterase 8,  zinc finger, SWIM-type containing 3,  spermatogenesis associated 25,  neuralized E3 ubiquitin protein ligase 2,  cathepsin A,  PDX1 C-terminal inhibiting factor 1, zinc finger protein 335 |

Additional file 4. continued

| ECA | Position | Number of SNPs in shared parts of ROH regions | Size of shared ROH (bp) | Gene ID | Human ortholog | Gene name/ function |
| --- | --- | --- | --- | --- | --- | --- |
| 22 | 36375683-36408439 | 57 | 32757 | ENSECAT00000015564 | *SULF2* | sulfatase 2 |
| 22 | 36540162-36580636 | 93 | 40475 | - | *-* | - |
| 22 | 38921253-38921253 | 1 | 1 | - | *-* | - |
| 22 | 39007445-39075522 | 127 | 68078 | - | *-* | - |
| 22 | 39252641-39296032 | 134 | 43392 | ENSECAT00000020322 | *NFATC2* | nuclear factor of activated T-cells, cytoplasmic, calcineurin-dependent 2 |
| 22 | 39494036-39542955 | 160 | 48920 | ENSECAT00000023315 | *ATP9A* | ATPase, class II, type 9A |
| 22 | 40334092-40380702 | 282 | 46611 | - | *-* | - |
| 22 | 40434540-40540812 | 496 | 106273 | - | *-* | - |
| 22 | 41084770-41084770 | 1 | 1 | - | *-* | - |
| 22 | 41409954-41457833 | 266 | 47880 | ENSECAT00000017433 | *CYP24A1* | cytochrome P450, family 24, subfamily A, polypeptide 1 |
| 22 | 41652351-41671837 | 90 | 19487 | - | *-* | - |
| 22 | 41995581-42050580 | 227 | 55000 | - | *-* | - |
| 22 | 42240838-42242401 | 7 | 1564 | - | *-* | - |
| 22 | 46427032-46427038 | 2 | 7 | - | *-* | - |
| 23 | 4156065-4168390 | 59 | 12326 | ENSECAT00000013155, ENSECAT00000013168 | *C9ORF153, C9ORF153* | chromosome 9 open reading frame 153 |
| 23 | 4241944-4242240 | 10 | 297 | - | *-* | - |
| 23 | 7670845-7722517 | 455 | 51673 | - | *-* | - |

Additional file 4. continued

| ECA | Position | Number of SNPs in shared parts of ROH regions | Size of shared ROH (bp) | Gene ID | Human ortholog | Gene name/ function |
| --- | --- | --- | --- | --- | --- | --- |
| 23 | 53327187-53327187 | 1 | 1 | - | *-* | - |
| 24 | 7306194-7306520 | 3 | 327 | ENSECAT00000016052 | *novel gene* |  |
| 24 | 17528700-17628242 | 744 | 99543 | ENSECAT00000026205, ENSECAT00000026207 | *novel gene,*  *SIPA1L1* | signal-induced proliferation-associated 1 like 1 |
| 24 | 25817714-25863994 | 187 | 46281 | - | *-* | - |
| 24 | 28384897-28485736 | 717 | 100840 | - | *-* | - |
| 24 | 28749715-28808854 | 331 | 59140 | - | *-* | - |
| 24 | 43672642-43722790 | 152 | 50149 | ENSECAT00000028966, ENSECAT00000017777 | *DYNC1H1, DYNC1H1* | dynein, cytoplasmic 1, heavy chain 1 |
| 26 | 6724950-6725629 | 12 | 680 | - | *-* | - |
| 26 | 8981339-9010868 | 289 | 29530 | - | *-* | - |
| 27 | 7296187-7308210 | 20 | 12024 | - | *-* | - |
| 27 | 7352090-7374133 | 41 | 22044 | ENSECAT00000017018, ENSECAT00000017020 | *FGFR1,*  *FGFR1* | fibroblast growth factor receptor 1 |
| 28 | 4769017-4769850 | 12 | 834 | ENSECAT00000007163 | *ZDHHC17* | zinc finger, DHHC-type containing 17 |
| 28 | 4770539-4770808 | 3 | 270 | ENSECAT00000007163 | *ZDHHC17* | zinc finger, DHHC-type containing 17 |
| 28 | 7677063-7677245 | 6 | 183 | ENSECAT00000010236 | *OTOGL* | otogelin-like |
| 28 | 8124581-8173006 | 190 | 48426 | ENSECAT00000003046 | *LIN7A* | lin-7 homolog A (C. elegans) |

Additional file 4. continued

| ECA | Position | Number of SNPs in shared parts of ROH regions | Size of shared ROH (bp) | Gene ID | Human ortholog | Gene name/ function |
| --- | --- | --- | --- | --- | --- | --- |
| 28 | 8174301-8174967 | 9 | 667 | ENSECAT00000003046, ENSECAT00000008292 | *LIN7A,*  *novel gene* | lin-7 homolog A (C. elegans) |
| 28 | 8319544-8394085 | 285 | 74542 | ENSECAT00000008574 | *ACSS3* | acyl-CoA synthetase short-chain family member 3 |
| 28 | 8394510-8604682 | 964 | 210173 | ENSECAT00000008574 | *ACSS3* | acyl-CoA synthetase short-chain family member 3 |
| 28 | 8669741-8970963 | 1381 | 301223 | ENSECAT00000012983, ENSECAT00000012992 | *PPFIA2,*  *PPFIA2* | protein tyrosine phosphatase, receptor type, f polypeptide (PTPRF), interacting protein (liprin), alpha 2 |
| 28 | 8971186-9066771 | 616 | 95586 | ENSECAT00000012983, ENSECAT00000012992 | *PPFIA2,*  *PPFIA2* | protein tyrosine phosphatase, receptor type, F polypeptide (PTPRF), interacting protein (liprin), alpha 2 |
| 28 | 9268372-9276841 | 81 | 8470 | ENSECAT00000010706 | *novel gene* |  |
| 28 | 10029782-10029883 | 5 | 102 | ENSECAT00000015432 | *TMTC2* | transmembrane and tetratricopeptide repeat containing 2 |
| 28 | 11828267-11828821 | 19 | 555 | - | *-* | - |
| 28 | 11916379-11916416 | 4 | 38 | - | *-* | - |
| 28 | 12852791-12852804 | 3 | 14 | - | *-* | - |

Additional file 4. continued

| ECA | Position | Number of SNPs in shared parts of ROH regions | Size of shared ROH (bp) | Gene ID | Human ortholog | Gene name/ function |
| --- | --- | --- | --- | --- | --- | --- |
| 28 | 12945134-12945134 | 1 | 1 | - | *-* | - |
| 28 | 16360254-16360269 | 3 | 16 | - | *-* | - |
| 28 | 17863936-17863937 | 2 | 2 | ENSECAT00000022601 | *novel gene* |  |
| 28 | 34955841-34987054 | 21 | 31214 | ENSECAT00000007128, ENSECAT00000009152, ENSECAT00000026801, ENSECAT00000012534, ENSECAT00000012539 | *CDC42EP1, LGALS2,*  *ANKRD54,*  *TRIOBP,*  *TRIOBP* | CDC42 effector protein (Rho GTPase binding) 1,  lectin, galactoside-binding, soluble, 2,  ankyrin repeat domain 54,  TRIO and F-actin binding protein |
| 28 | 35140321-35208630 | 66 | 68310 | ENSECAT00000025369, ENSECAT00000025987 | *POLR2F,*  *SOX10* | polymerase (RNA) II (DNA directed) polypeptide F,  SRY (sex determining region Y)-box 10 |
| 29 | 21025984-21068597 | 127 | 42614 | ENSECAT00000014158, ENSECAT00000014180 | *FRMD4A,*  *FRMD4A* | FERM domain containing 4A |
| 29 | 31013363-31053420 | 224 | 40058 | - | *-* | - |
| 29 | 31322916-31395846 | 489 | 72931 | - | *-* | - |
| 31 | 655869-655869 | 1 | 1 | - | *-* | - |

Additional file 4. continued

| ECA | Position | Number of SNPs in shared parts of ROH regions | Size of shared ROH (bp) | Gene ID | Human ortholog | Gene name/ function |
| --- | --- | --- | --- | --- | --- | --- |
| **500-SNP window** |  |  |  |  |  |  |
| 22 | 35093301-35093301 | 2 | 1 | - | *-* | - |
| 22 | 35912192-35912253 | 8 | 62 | ENSECAT00000023571 | EYA2 | EYA transcriptional coactivator and phosphatase 2 |
| 22 | 36375683-36408439 | 57 | 32757 | ENSECAT00000015564 | SULF2 | sulfatase 2 |
| 28 | 8394510-8970963 | 2647 | 576454 | ENSECAT00000008574, ENSECAT00000012983,  ENSECAT00000012992,  ENSECAT00000029643 | ACSS3,  PPFIA2,  PPFIA2,  RNA5SP220 | acyl-CoA synthetase short-chain family member 3,  protein tyrosine phosphatase, receptor type, F polypeptide (PTPRF), interacting protein (liprin), alpha 2,  RNA, 5S ribosomal pseudogene 220 |
| 28 | 10029782-10089734 | 324 | 59953 | ENSECAT00000015432 | *TMTC2* | transmembrane and tetratricopeptide repeat containing 2 |
| 28 | 15007687-15099149 | 296 | 91463 | - | *-* | - |
| 28 | 17864172-18000136 | 681 | 135965 | ENSECAT00000022601 | *novel gene* |  |
