# Supplementary material for: Runs of homozygosity reveal signatures of positive selection for reproduction traits in breed and non-breed horses
Source: BMC Genomics. 2015 Oct 9;16:764. doi: 10.1186/s12864-015-1977-3 (PMC4600213; doi:10.1186/s12864-015-1977-3)
Supplement: Additional file 5: — Shared runs of homozygosity (ROHs) in breed horses. The consensus private ROH regions and genes of Hanoverian, Arabian, Saxon-Thuringian Heavy Warmblood and Thoroughbred horses in 50-SNP windows are shown. The number of SNPs and size of shared ROH indicate the overlap of homozygous variants. (DOCX 14 kb) [file 12864_2015_1977_MOESM5_ESM.docx]

Additional file 5. Shared runs of homozygosity (ROHs) in breed horses. The consensus private ROH regions and genes of Hanoverian, Arabian, Saxon-Thuringian Heavy Warmblood and Thoroughbred horses in 50-SNP windows are shown. The number of SNPs and size of shared ROH indicate the overlap of homozygous variants.

| ECA | Position | Number of SNPs in shared parts of ROH regions | Size of shared ROH (bp) | Gene ID | Human ortholog | Gene name/ function |
| --- | --- | --- | --- | --- | --- | --- |
| **50-SNP window** |  |  |  |  |  |  |
| 6 | 2281286- 2352627 | 283 | 71342 | - | *-* | - |
| 8 | 41252672-41307411 | 128 | 54740 | ENSECAG00000021666 | YES1 | V-Yes-1 Yamaguchi Sarcoma Viral Oncogene Homolog 1 |
| 8 | 25907764-25907831 | 6 | 68 | - | *-* | - |
