# Supplementary material for: Runs of homozygosity reveal signatures of positive selection for reproduction traits in breed and non-breed horses
Source: BMC Genomics. 2015 Oct 9;16:764. doi: 10.1186/s12864-015-1977-3 (PMC4600213; doi:10.1186/s12864-015-1977-3)
Supplement: Additional file 7: — Mutations with high or moderate effects in ROHs (50-SNP windows) of non-breed horses. The ROH position and size (EquCab2.70), the position of SNPs, their mutant allele, potential impact and type are shown. Impact estimations are derived from SNPEff predictions. (DOCX 65 kb) [file 12864_2015_1977_MOESM7_ESM.docx]

Additional file 7. Mutations with high or moderate effects in ROHs (50-SNP windows) of non-breed horses. The ROH position and size (EquCab2.70), the position of SNPs, their mutant allele, potential impact and type are shown. Impact estimations are derived from SNPEff predictions.

| Chromosome | ROH start | ROH end | Size | Position | | Reference allele | | Mutant allele | | Impact | Type | Gene | Transcript |
| --- | --- | --- | --- | --- | --- | --- | --- | --- | --- | --- | --- | --- | --- |
| 1 | 129290715 | 129374751 | 84037 | 129368720 | | A | | C | | MODERATE | MISSENSE_VARIANT | TLN2 | ENSECAT00000023339 |
| 1 | 129290715 | 129374751 | 84037 | 129368720 | | A | | C | | MODERATE | MISSENSE_VARIANT | TLN2 | ENSECAT00000023447 |
| 1 | 129290715 | 129374751 | 84037 | 129368720 | | A | | C | | MODERATE | MISSENSE_VARIANT | TLN2 | ENSECAT00000023476 |
| 1 | 129934428 | 130014163 | 79736 | 129943691 | | T | | C | | MODERATE | MISSENSE_VARIANT | VPS13C | ENSECAT00000019241 |
| 1 | 129934428 | 130014163 | 79736 | 130002899 | | T | | C | | MODERATE | MISSENSE_VARIANT | VPS13C | ENSECAT00000019241 |
| 1 | 129934428 | 130014163 | 79736 | 130002908 | | C | | G | | MODERATE | MISSENSE_VARIANT&  SPLICE_REGION_VARIANT | VPS13C | ENSECAT00000019241 |
| 1 | 130054318 | 130129275 | 74958 | 130076737 | | A | | G | | MODERATE | MISSENSE_VARIANT | VPS13C | ENSECAT00000019241 |
| 2 | 79888517 | 80111836 | 223320 | 80064991 | | T | | C | | MODERATE | MISSENSE_VARIANT | SFRP2 | ENSECAT00000018012 |
| 2 | 79888517 | 80111836 | 223320 | 80064992 | | T | | C | | MODERATE | MISSENSE_VARIANT | SFRP2 | ENSECAT00000018012 |
| 2 | 79888517 | 80111836 | 223320 | 80065013 | | T | | C | | MODERATE | MISSENSE_VARIANT | SFRP2 | ENSECAT00000018012 |
| 3 | 58133505 | 58337269 | 203765 | 58152162 | | T | | A | | MODERATE | MISSENSE_VARIANT | FRAS1 | ENSECAT00000008119 |
| 3 | 58133505 | 58337269 | 203765 | 58153643 | | C | | T | | MODERATE | MISSENSE_VARIANT | FRAS1 | ENSECAT00000008119 |
| 3 | 58133505 | 58337269 | 203765 | 58164196 | | A | | G | | MODERATE | MISSENSE_VARIANT | FRAS1 | ENSECAT00000008119 |
| 3 | 58133505 | 58337269 | 203765 | 58164302 | | C | | A | | MODERATE | MISSENSE_VARIANT | FRAS1 | ENSECAT00000008119 |
| 4 | 39776894 | 39832480 | 55587 | 39830948 | | A | | G | | MODERATE | MISSENSE_VARIANT | ENSECAG00000001311 | ENSECAT00000001132 |
| 4 | 39776894 | 39832480 | 55587 | 39830966 | | G | | C | | MODERATE | MISSENSE_VARIANT | ENSECAG00000001311 | ENSECAT00000001132 |
| 7 | 85593736 | 85620317 | 26582 | 85609847 | | C | | T | | MODERATE | MISSENSE_VARIANT | NUCB2 | ENSECAT00000017959 |
| 7 | 85593736 | 85620317 | 26582 | 85609847 | | C | | T | | MODERATE | MISSENSE_VARIANT | NUCB2 | ENSECAT00000017896 |
| 9 | 65689063 | 65773640 | 84578 | 65693045 | | G | | A | | MODERATE | MISSENSE_VARIANT | FAM83A | ENSECAT00000020108 |
| 9 | 65689063 | 65773640 | 84578 | 65711418 | | A | | G | | MODERATE | MISSENSE_VARIANT | FAM83A | ENSECAT00000020108 |
|  |  |  |  | |  | |  | |  |  |  |  |  |
|  |  |  |  | |  | |  | |  |  |  |  |  |
|  |  |  |  | |  | |  | |  |  |  |  |  |
|  |  |  |  | |  | |  | |  |  |  |  |  |
| Additional file 7. continued | | | | |  | |  | |  |  |  |  |  |
| chromosome | ROH start | ROH end | size | | position | | reference allele | | mutant allele | impact | type | gene | transcript |
| 9 | 65689063 | 65773640 | 84578 | | 65711502 | | A | | G | MODERATE | MISSENSE_VARIANT | FAM83A | ENSECAT00000020108 |
| 9 | 65689063 | 65773640 | 84578 | | 65711802 | | T | | C | MODERATE | MISSENSE_VARIANT | FAM83A | ENSECAT00000020108 |
| 9 | 65689063 | 65773640 | 84578 | | 65729754 | | T | | C | MODERATE | MISSENSE_VARIANT | ENSECAG00000020843 | ENSECAT00000022212 |
| 9 | 65689063 | 65773640 | 84578 | | 65734559 | | C | | T | MODERATE | MISSENSE_VARIANT | ENSECAG00000020843 | ENSECAT00000022212 |
| 10 | 14630027 | 14653098 | 23072 | | 14639454 | | A | | G | MODERATE | MISSENSE_VARIANT | PLAUR | ENSECAT00000023704 |
| 10 | 14630027 | 14653098 | 23072 | | 14639516 | | A | | G | MODERATE | MISSENSE_VARIANT | PLAUR | ENSECAT00000023704 |
| 10 | 14630027 | 14653098 | 23072 | | 14639543 | | A | | G | MODERATE | MISSENSE_VARIANT | PLAUR | ENSECAT00000023704 |
| 10 | 14630027 | 14653098 | 23072 | | 14645199 | | T | | C | MODERATE | MISSENSE_VARIANT | PLAUR | ENSECAT00000023704 |
| 10 | 14630027 | 14653098 | 23072 | | 14648550 | | T | | C | MODERATE | MISSENSE_VARIANT | PLAUR | ENSECAT00000023704 |
| 10 | 15782483 | 15858844 | 76362 | | 15794439 | | C | | A | MODERATE | MISSENSE_VARIANT | ENSECAG00000023091 | ENSECAT00000024682 |
| 10 | 15782483 | 15858844 | 76362 | | 15795240 | | A | | G | MODERATE | MISSENSE_VARIANT | ENSECAG00000023091 | ENSECAT00000024682 |
| 10 | 15782483 | 15858844 | 76362 | | 15810284 | | G | | A | MODERATE | MISSENSE_VARIANT | ENSECAG00000023091 | ENSECAT00000024682 |
| 10 | 15782483 | 15858844 | 76362 | | 15828475 | | C | | T | MODERATE | MISSENSE_VARIANT | EXOC3L2 | ENSECAT00000025644 |
| 10 | 15782483 | 15858844 | 76362 | | 15828563 | | T | | C | MODERATE | MISSENSE_VARIANT | EXOC3L2 | ENSECAT00000025644 |
| 10 | 15782483 | 15858844 | 76362 | | 15829197 | | T | | C | MODERATE | MISSENSE_VARIANT | EXOC3L2 | ENSECAT00000025644 |
| 10 | 15782483 | 15858844 | 76362 | | 15829899 | | G | | A | MODERATE | MISSENSE_VARIANT | EXOC3L2 | ENSECAT00000025644 |
| 10 | 15782483 | 15858844 | 76362 | | 15832860 | | T | | C | MODERATE | MISSENSE_VARIANT | EXOC3L2 | ENSECAT00000025644 |
| 10 | 15782483 | 15858844 | 76362 | | 15832878 | | T | | C | MODERATE | MISSENSE_VARIANT | EXOC3L2 | ENSECAT00000025644 |
| 10 | 15782483 | 15858844 | 76362 | | 15837614 | | C | | T | MODERATE | MISSENSE_VARIANT | EXOC3L2 | ENSECAT00000025644 |
| 10 | 15782483 | 15858844 | 76362 | | 15854304 | | T | | C | MODERATE | MISSENSE_VARIANT | MARK4 | ENSECAT00000000882 |
| 10 | 15782483 | 15858844 | 76362 | | 15854304 | | T | | C | MODERATE | MISSENSE_VARIANT | MARK4 | ENSECAT00000001234 |
| 10 | 15782483 | 15858844 | 76362 | | 15855337 | | A | | G | HIGH | SPLICE_ACCEPTOR_VARIANT  &INTRON_VARIANT | MARK4 | ENSECAT00000000882 |
| 10 | 15782483 | 15858844 | 76362 | | 15855337 | | A | | G | HIGH | SPLICE_ACCEPTOR_VARIANT  &INTRON_VARIANT | MARK4 | ENSECAT00000001234 |
| 10 | 17034967 | 17104150 | 69184 | | 17085282 | | A | | G | MODERATE | MISSENSE_VARIANT | ARHGAP35 | ENSECAT00000018888 |
| 10 | 19028284 | 19069077 | 40794 | | 19028291 | | C | | A | MODERATE | MISSENSE_VARIANT | PPFIA3 | ENSECAT00000004206 |
| Additional file 7. continued | | | | |  | |  | |  |  |  |  |  |
| chromosome | ROH start | ROH end | size | | position | | reference allele | | mutant allele | impact | type | gene | transcript |
| 10 | 19028284 | 19069077 | 40794 | | 19034666 | | C | | G | MODERATE | MISSENSE_VARIANT | HRC | ENSECAT00000021257 |
| 10 | 19028284 | 19069077 | 40794 | | 19035572 | | C | | A | MODERATE | MISSENSE_VARIANT | HRC | ENSECAT00000021257 |
| 10 | 19028284 | 19069077 | 40794 | | 19035729 | | C | | G | MODERATE | MISSENSE_VARIANT | HRC | ENSECAT00000021257 |
| 10 | 19028284 | 19069077 | 40794 | | 19036735 | | C | | T | MODERATE | MISSENSE_VARIANT | HRC | ENSECAT00000021257 |
| 10 | 19028284 | 19069077 | 40794 | | 19036757 | | C | | T | MODERATE | MISSENSE_VARIANT | HRC | ENSECAT00000021257 |
| 10 | 19028284 | 19069077 | 40794 | | 19037152 | | C | | T | MODERATE | MISSENSE_VARIANT | HRC | ENSECAT00000021257 |
| 10 | 19028284 | 19069077 | 40794 | | 19037159 | | A | | G | MODERATE | MISSENSE_VARIANT | HRC | ENSECAT00000021257 |
| 10 | 19028284 | 19069077 | 40794 | | 19037561 | | A | | G | MODERATE | MISSENSE_VARIANT | HRC | ENSECAT00000021257 |
| 10 | 19028284 | 19069077 | 40794 | | 19057302 | | G | | A | MODERATE | MISSENSE_VARIANT | TRPM4 | ENSECAT00000023252 |
| 10 | 19028284 | 19069077 | 40794 | | 19062696 | | G | | A | MODERATE | MISSENSE_VARIANT | TRPM4 | ENSECAT00000023252 |
| 10 | 20896796 | 20961513 | 64718 | | 20918519 | | G | | A | MODERATE | MISSENSE_VARIANT  &SPLICE_REGION_  VARIANT | ENSECAG00000023610 | ENSECAT00000025272 |
| 10 | 20896796 | 20961513 | 64718 | | 20918576 | | C | | T | MODERATE | MISSENSE_VARIANT | ENSECAG00000023610 | ENSECAT00000025272 |
| 10 | 20896796 | 20961513 | 64718 | | 20919525 | | T | | A | MODERATE | MISSENSE_VARIANT | ENSECAG00000023610 | ENSECAT00000025272 |
| 10 | 20896796 | 20961513 | 64718 | | 20919612 | | G | | C | MODERATE | MISSENSE_VARIANT | ENSECAG00000023610 | ENSECAT00000025272 |
| 10 | 20896796 | 20961513 | 64718 | | 20931484 | | C | | T | MODERATE | MISSENSE_VARIANT | SIGLECL1 | ENSECAT00000025953 |
| 10 | 20896796 | 20961513 | 64718 | | 20933211 | | G | | C | MODERATE | MISSENSE_VARIANT | SIGLECL1 | ENSECAT00000025953 |
| 10 | 22165158 | 22227140 | 61983 | | 22177667 | | G | | A | MODERATE | MISSENSE_VARIANT | ENSECAG00000000992 | ENSECAT00000000805 |
| 10 | 22165158 | 22227140 | 61983 | | 22177897 | | G | | A | MODERATE | MISSENSE_VARIANT | ENSECAG00000000992 | ENSECAT00000000805 |
| 10 | 22165158 | 22227140 | 61983 | | 22178052 | | T | | G | MODERATE | MISSENSE_VARIANT | ENSECAG00000000992 | ENSECAT00000000805 |
| 10 | 22165158 | 22227140 | 61983 | | 22190014 | | G | | T | HIGH | STOP_GAINED | EQUCABV1R921 | ENSECAT00000000844 |
| 10 | 22165158 | 22227140 | 61983 | | 22190295 | | A | | T | MODERATE | MISSENSE_VARIANT | EQUCABV1R921 | ENSECAT00000000844 |
| 10 | 22165158 | 22227140 | 61983 | | 22190539 | | A | | G | MODERATE | MISSENSE_VARIANT | EQUCABV1R921 | ENSECAT00000000844 |
| 10 | 22165158 | 22227140 | 61983 | | 22190636 | | G | | T | MODERATE | MISSENSE_VARIANT | EQUCABV1R921 | ENSECAT00000000844 |
| 10 | 22165158 | 22227140 | 61983 | | 22202244 | | C | | A | MODERATE | MISSENSE_VARIANT | EQUCABV1R920 | ENSECAT00000000869 |
| 10 | 22165158 | 22227140 | 61983 | | 22202542 | | T | | C | MODERATE | MISSENSE_VARIANT | EQUCABV1R920 | ENSECAT00000000869 |
| 10 | 22165158 | 22227140 | 61983 | | 22202565 | | C | | A | MODERATE | MISSENSE_VARIANT | EQUCABV1R920 | ENSECAT00000000869 |
| Additional file 7. continued | | | | |  | |  | |  |  |  |  |  |
| chromosome | ROH start | ROH end | size | | position | | reference allele | | mutant allele | impact | type | gene | transcript |
| 10 | 22165158 | 22227140 | 61983 | | 22202621 | | G | | C | MODERATE | MISSENSE_VARIANT | EQUCABV1R920 | ENSECAT00000000869 |
| 10 | 22165158 | 22227140 | 61983 | | 22202635 | | C | | T | MODERATE | MISSENSE_VARIANT | EQUCABV1R920 | ENSECAT00000000869 |
| 10 | 22165158 | 22227140 | 61983 | | 22202764 | | G | | A | MODERATE | MISSENSE_VARIANT | EQUCABV1R920 | ENSECAT00000000869 |
| 10 | 22165158 | 22227140 | 61983 | | 22219523 | | A | | G | MODERATE | MISSENSE_VARIANT | EQUCABV1R919 | ENSECAT00000000936 |
| 10 | 22165158 | 22227140 | 61983 | | 22219700 | | C | | T | MODERATE | MISSENSE_VARIANT | EQUCABV1R919 | ENSECAT00000000936 |
| 10 | 22165158 | 22227140 | 61983 | | 22219725 | | T | | C | MODERATE | MISSENSE_VARIANT | EQUCABV1R919 | ENSECAT00000000936 |
| 10 | 22165158 | 22227140 | 61983 | | 22220000 | | A | | G | MODERATE | MISSENSE_VARIANT | EQUCABV1R919 | ENSECAT00000000936 |
| 10 | 22165158 | 22227140 | 61983 | | 22220172 | | G | | T | MODERATE | MISSENSE_VARIANT | EQUCABV1R919 | ENSECAT00000000936 |
| 10 | 22228721 | 22283734 | 55014 | | 22246029 | | C | | T | MODERATE | MISSENSE_VARIANT | ENSECAG00000023102 | ENSECAT00000024688 |
| 10 | 32049398 | 32133977 | 84580 | | 32086489 | | C | | T | MODERATE | MISSENSE_VARIANT | MEI4 | ENSECAT00000007041 |
| 10 | 32049398 | 32133977 | 84580 | | 32086524 | | T | | C | HIGH | SPLICE_DONOR_  VARIANT  &INTRON_VARIANT | MEI4 | ENSECAT00000007041 |
| 10 | 32049398 | 32133977 | 84580 | | 32117180 | | C | | T | MODERATE | MISSENSE_VARIANT | MEI4 | ENSECAT00000007041 |
| 10 | 33069658 | 33070748 | 1091 | | 33070457 | | C | | T | MODERATE | MISSENSE_VARIANT  &SPLICE_REGION_  VARIANT | ENSECAG00000008305 | ENSECAT00000008375 |
| 10 | 33069658 | 33070748 | 1091 | | 33070684 | | C | | A | MODERATE | MISSENSE_VARIANT | ENSECAG00000008305 | ENSECAT00000008375 |
| 10 | 33069658 | 33070748 | 1091 | | 33070730 | | G | | T | MODERATE | MISSENSE_VARIANT | ENSECAG00000008305 | ENSECAT00000008375 |
| 10 | 33069658 | 33070748 | 1091 | | 33070748 | | T | | G | MODERATE | MISSENSE_VARIANT | ENSECAG00000008305 | ENSECAT00000008375 |
| 10 | 33137125 | 33461831 | 324707 | | 33191911 | | G | | A | MODERATE | MISSENSE_VARIANT | IRAK1BP1 | ENSECAT00000009409 |
| 10 | 33137125 | 33461831 | 324707 | | 33265715 | | C | | T | MODERATE | MISSENSE_VARIANT | PHIP | ENSECAT00000011576 |
| 10 | 34152280 | 34237984 | 85705 | | 34179092 | | C | | T | MODERATE | MISSENSE_VARIANT | ELOVL4 | ENSECAT00000024536 |
| 10 | 34152280 | 34237984 | 85705 | | 34221357 | | G | | A | MODERATE | MISSENSE_VARIANT | TTK | ENSECAT00000025743 |
| 11 | 8384586 | 8421188 | 36603 | | 8405695 | | G | | C | MODERATE | MISSENSE_VARIANT | COG1 | ENSECAT00000008658 |
| 13 | 32790136 | 32887855 | 97720 | | 32798351 | | C | | G | MODERATE | MISSENSE_VARIANT | ZC3H7A | ENSECAT00000005954 |
| 13 | 32790136 | 32887855 | 97720 | | 32816037 | | A | | G | MODERATE | MISSENSE_VARIANT | ZC3H7A | ENSECAT00000005954 |
| 13 | 32790136 | 32887855 | 97720 | | 32820572 | | A | | G | MODERATE | MISSENSE_VARIANT | TXNDC11 | ENSECAT00000014164 |
|  |  |  |  | |  | |  | |  |  |  |  |  |
| Additional file 7. continued | | | | |  | |  | |  |  |  |  |  |
| chromosome | ROH start | ROH end | size | | position | | reference allele | | mutant allele | impact | type | gene | transcript |
| 13 | 32790136 | 32887855 | 97720 | | 32820586 | | A | | G | MODERATE | MISSENSE_VARIANT | TXNDC11 | ENSECAT00000014164 |
| 13 | 32790136 | 32887855 | 97720 | | 32820590 | | A | | G | MODERATE | MISSENSE_VARIANT | TXNDC11 | ENSECAT00000014164 |
| 13 | 32790136 | 32887855 | 97720 | | 32820592 | | A | | G | MODERATE | MISSENSE_VARIANT | TXNDC11 | ENSECAT00000014164 |
| 13 | 32790136 | 32887855 | 97720 | | 32820613 | | A | | T | MODERATE | MISSENSE_VARIANT | TXNDC11 | ENSECAT00000014164 |
| 13 | 32790136 | 32887855 | 97720 | | 32820623 | | A | | G | MODERATE | MISSENSE_VARIANT  &SPLICE_REGION_  VARIANT | TXNDC11 | ENSECAT00000014164 |
| 13 | 32790136 | 32887855 | 97720 | | 32864367 | | T | | C | MODERATE | MISSENSE_VARIANT | TXNDC11 | ENSECAT00000014164 |
| 13 | 32790136 | 32887855 | 97720 | | 32864574 | | T | | C | MODERATE | MISSENSE_VARIANT | TXNDC11 | ENSECAT00000014164 |
| 13 | 32790136 | 32887855 | 97720 | | 32864588 | | G | | C | MODERATE | MISSENSE_VARIANT | TXNDC11 | ENSECAT00000014164 |
| 13 | 32790136 | 32887855 | 97720 | | 32864721 | | C | | G | MODERATE | MISSENSE_VARIANT  &SPLICE_REGION_  VARIANT | TXNDC11 | ENSECAT00000014164 |
| 21 | 49837032 | 49951766 | 114735 | | 49882847 | | G | | C | MODERATE | MISSENSE_VARIANT | TAS2R1 | ENSECAT00000005035 |
| 21 | 50023234 | 50119009 | 95776 | | 50116854 | | G | | A | MODERATE | MISSENSE_VARIANT | SEMA5A | ENSECAT00000017338 |
| 21 | 50270718 | 50330925 | 60208 | | 50302914 | | T | | G | MODERATE | MISSENSE_VARIANT | SEMA5A | ENSECAT00000017338 |
| 21 | 50270718 | 50330925 | 60208 | | 50302915 | | G | | C | MODERATE | MISSENSE_VARIANT | SEMA5A | ENSECAT00000017338 |
| 22 | 34023768 | 34136641 | 112874 | | 34086087 | | A | | G | MODERATE | MISSENSE_VARIANT | PABPC1L | ENSECAT00000024818 |
| 22 | 34023768 | 34136641 | 112874 | | 34086614 | | C | | A | MODERATE | MISSENSE_VARIANT | PABPC1L | ENSECAT00000024818 |
| 22 | 34023768 | 34136641 | 112874 | | 34086823 | | T | | A | MODERATE | MISSENSE_VARIANT | PABPC1L | ENSECAT00000024818 |
| 22 | 34023768 | 34136641 | 112874 | | 34086838 | | A | | G | MODERATE | MISSENSE_VARIANT | PABPC1L | ENSECAT00000024818 |
| 22 | 34023768 | 34136641 | 112874 | | 34087464 | | C | | T | MODERATE | MISSENSE_VARIANT | PABPC1L | ENSECAT00000024818 |
| 22 | 34023768 | 34136641 | 112874 | | 34105691 | | T | | C | MODERATE | MISSENSE_VARIANT | TOMM34 | ENSECAT00000021700 |
| 22 | 34023768 | 34136641 | 112874 | | 34110139 | | A | | G | MODERATE | MISSENSE_VARIANT | TOMM34 | ENSECAT00000021700 |
| 22 | 34023768 | 34136641 | 112874 | | 34110154 | | T | | C | MODERATE | MISSENSE_VARIANT | TOMM34 | ENSECAT00000021700 |
| 22 | 34023768 | 34136641 | 112874 | | 34110160 | | A | | T | MODERATE | MISSENSE_VARIANT | TOMM34 | ENSECAT00000021700 |
| 22 | 34136970 | 34241197 | 104228 | | 34216841 | | C | | T | MODERATE | MISSENSE_VARIANT | KCNS1 | ENSECAT00000010717 |
|  |  |  |  | |  | |  | |  |  |  |  |  |
|  |  |  |  | |  | |  | |  |  |  |  |  |
| Additional file 7. continued | | | | |  | |  | |  |  |  |  |  |
| chromosome | ROH start | ROH end | size | | position | | reference allele | | mutant allele | impact | type | gene | transcript |
| 22 | 34136970 | 34241197 | 104228 | | 34220281 | | C | | G | HIGH | SPLICE_DONOR_  VARIANT  &SPLICE_REGION_  VARIANT  &INTRON_VARIANT | KCNS1 | ENSECAT00000010717 |
| 22 | 34136970 | 34241197 | 104228 | | 34220339 | | G | | C | MODERATE | MISSENSE_VARIANT | KCNS1 | ENSECAT00000010717 |
| 22 | 34136970 | 34241197 | 104228 | | 34220347 | | T | | G | MODERATE | MISSENSE_VARIANT | KCNS1 | ENSECAT00000010717 |
| 22 | 34136970 | 34241197 | 104228 | | 34220349 | | T | | C | MODERATE | MISSENSE_VARIANT | KCNS1 | ENSECAT00000010717 |
| 22 | 34136970 | 34241197 | 104228 | | 34220373 | | T | | G | MODERATE | MISSENSE_VARIANT | KCNS1 | ENSECAT00000010717 |
| 22 | 34136970 | 34241197 | 104228 | | 34220467 | | C | | T | MODERATE | MISSENSE_VARIANT | KCNS1 | ENSECAT00000010717 |
| 22 | 34768055 | 34931170 | 163116 | | 34771510 | | C | | A | MODERATE | MISSENSE_VARIANT | DNTTIP1 | ENSECAT00000025003 |
| 22 | 34768055 | 34931170 | 163116 | | 34792343 | | C | | G | MODERATE | MISSENSE_VARIANT | DNTTIP1 | ENSECAT00000025003 |
| 22 | 34768055 | 34931170 | 163116 | | 34807271 | | G | | A | MODERATE | MISSENSE_VARIANT | SNX21 | ENSECAT00000023028 |
| 22 | 34768055 | 34931170 | 163116 | | 34807852 | | A | | G | MODERATE | MISSENSE_VARIANT | SNX21 | ENSECAT00000023028 |
| 22 | 34768055 | 34931170 | 163116 | | 34813923 | | G | | A | MODERATE | MISSENSE_VARIANT | ACOT8 | ENSECAT00000023435 |
| 22 | 34768055 | 34931170 | 163116 | | 34842006 | | G | | A | MODERATE | MISSENSE_VARIANT | ZSWIM3 | ENSECAT00000001514 |
| 22 | 34768055 | 34931170 | 163116 | | 34845200 | | A | | G | MODERATE | MISSENSE_VARIANT | ZSWIM1 | ENSECAT00000001375 |
| 22 | 34768055 | 34931170 | 163116 | | 34845599 | | C | | A | MODERATE | MISSENSE_VARIANT | ZSWIM1 | ENSECAT00000001375 |
| 22 | 34768055 | 34931170 | 163116 | | 34848133 | | T | | C | MODERATE | MISSENSE_VARIANT | SPATA25 | ENSECAT00000001682 |
| 22 | 34768055 | 34931170 | 163116 | | 34848356 | | G | | A | MODERATE | MISSENSE_VARIANT | SPATA25 | ENSECAT00000001682 |
| 22 | 34768055 | 34931170 | 163116 | | 34854965 | | G | | A | MODERATE | MISSENSE_VARIANT | CTSA | ENSECAT00000005323 |
| 22 | 34768055 | 34931170 | 163116 | | 34860108 | | G | | A | MODERATE | MISSENSE_VARIANT | PLTP | ENSECAT00000021279 |
| 22 | 34768055 | 34931170 | 163116 | | 34860288 | | C | | G | MODERATE | MISSENSE_VARIANT | PLTP | ENSECAT00000021279 |
| 22 | 34768055 | 34931170 | 163116 | | 34866791 | | C | | A | MODERATE | MISSENSE_VARIANT | PLTP | ENSECAT00000021279 |
| 22 | 34768055 | 34931170 | 163116 | | 34884898 | | C | | T | MODERATE | MISSENSE_VARIANT | PCIF1 | ENSECAT00000020342 |
| 22 | 34768055 | 34931170 | 163116 | | 34884898 | | C | | T | MODERATE | MISSENSE_VARIANT | PCIF1 | ENSECAT00000020385 |
| 22 | 34768055 | 34931170 | 163116 | | 34911634 | | G | | C | MODERATE | MISSENSE_VARIANT | ZNF335 | ENSECAT00000025620 |
| 24 | 17528700 | 17628242 | 99543 | | 17550541 | | G | | A | MODERATE | MISSENSE_VARIANT | SIPA1L1 | ENSECAT00000026205 |
| 24 | 17528700 | 17628242 | 99543 | | 17550541 | | G | | A | MODERATE | MISSENSE_VARIANT | SIPA1L1 | ENSECAT00000026207 |
| Additional file 7. continued | | | | |  | |  | |  |  |  |  |  |
| chromosome | ROH start | ROH end | size | | position | | reference allele | | mutant allele | impact | type | gene | transcript |
| 24 | 17528700 | 17628242 | 99543 | | 17611618 | | A | | G | MODERATE | MISSENSE_VARIANT | SIPA1L1 | ENSECAT00000026205 |
| 24 | 17528700 | 17628242 | 99543 | | 17611618 | | A | | G | MODERATE | MISSENSE_VARIANT | SIPA1L1 | ENSECAT00000026207 |
| 24 | 43672642 | 43722790 | 50149 | | 43698105 | | G | | C | MODERATE | MISSENSE_VARIANT | DYNC1H1 | ENSECAT00000028966 |
| 24 | 43672642 | 43722790 | 50149 | | 43698105 | | G | | C | MODERATE | MISSENSE_VARIANT | DYNC1H1 | ENSECAT00000017777 |
| 24 | 43672642 | 43722790 | 50149 | | 43698120 | | T | | G | MODERATE | MISSENSE_VARIANT | DYNC1H1 | ENSECAT00000028966 |
| 24 | 43672642 | 43722790 | 50149 | | 43698120 | | T | | G | MODERATE | MISSENSE_VARIANT | DYNC1H1 | ENSECAT00000017777 |
| 24 | 43672642 | 43722790 | 50149 | | 43698138 | | G | | C | HIGH | SPLICE_ACCEPTOR_VARIANT  &SPLICE_REGION_  VARIANT&INTRON_VARIANT | DYNC1H1 | ENSECAT00000017777 |
| 24 | 43672642 | 43722790 | 50149 | | 43698148 | | A | | G | MODERATE | MISSENSE_VARIANT | DYNC1H1 | ENSECAT00000028966 |
| 24 | 43672642 | 43722790 | 50149 | | 43698148 | | A | | G | MODERATE | MISSENSE_VARIANT | DYNC1H1 | ENSECAT00000017777 |
| 24 | 43672642 | 43722790 | 50149 | | 43698150 | | A | | G | MODERATE | MISSENSE_VARIANT | DYNC1H1 | ENSECAT00000028966 |
| 24 | 43672642 | 43722790 | 50149 | | 43698150 | | A | | G | MODERATE | MISSENSE_VARIANT | DYNC1H1 | ENSECAT00000017777 |
| 24 | 43672642 | 43722790 | 50149 | | 43717701 | | A | | C | MODERATE | MISSENSE_VARIANT | DYNC1H1 | ENSECAT00000028966 |
| 24 | 43672642 | 43722790 | 50149 | | 43717701 | | A | | C | MODERATE | MISSENSE_VARIANT | DYNC1H1 | ENSECAT00000017777 |
| 24 | 43672642 | 43722790 | 50149 | | 43717735 | | A | | G | MODERATE | MISSENSE_VARIANT | DYNC1H1 | ENSECAT00000028966 |
| 24 | 43672642 | 43722790 | 50149 | | 43717735 | | A | | G | MODERATE | MISSENSE_VARIANT | DYNC1H1 | ENSECAT00000017777 |
| 27 | 7352090 | 7374133 | 22044 | | 7370666 | | G | | A | MODERATE | MISSENSE_VARIANT | LETM2 | ENSECAT00000025506 |
| 28 | 8319544 | 8394085 | 74542 | | 8377280 | | A | | G | MODERATE | MISSENSE_VARIANT | ACSS3 | ENSECAT00000008574 |
| 28 | 8319544 | 8394085 | 74542 | | 8377339 | | G | | C | MODERATE | MISSENSE_VARIANT | ACSS3 | ENSECAT00000008574 |
| 28 | 8394510 | 8604682 | 210173 | | 8441975 | | T | | C | MODERATE | MISSENSE_VARIANT  &SPLICE_REGION_  VARIANT | ACSS3 | ENSECAT00000008574 |
| 28 | 8394510 | 8604682 | 210173 | | 8489080 | | T | | C | MODERATE | MISSENSE_VARIANT | ACSS3 | ENSECAT00000008574 |
| 28 | 8394510 | 8604682 | 210173 | | 8584415 | | T | | G | MODERATE | MISSENSE_VARIANT | PPFIA2 | ENSECAT00000012983 |
| 28 | 8394510 | 8604682 | 210173 | | 8584415 | | T | | G | MODERATE | MISSENSE_VARIANT | PPFIA2 | ENSECAT00000012992 |
|  |  |  |  | |  | |  | |  |  |  |  |  |
| Additional file 7. continued | | | | |  | |  | |  |  |  |  |  |
| chromosome | ROH start | ROH end | size | | position | | reference allele | | mutant allele | impact | type | gene | transcript |
| 28 | 9268372 | 9276841 | 8470 | | 9276841 | | C | | T | HIGH | SPLICE_DONOR_  VARIANT  &SPLICE_REGION_  VARIANT  &INTRON_VARIANT | ENSECAG00000010438 | ENSECAT00000010706 |
| 28 | 17863936 | 17863937 | 2 | | 17863936 | | G | | T | MODERATE | MISSENSE_VARIANT | ENSECAG00000021273 | ENSECAT00000022601 |
| 28 | 17863936 | 17863937 | 2 | | 17863937 | | T | | A | MODERATE | MISSENSE_VARIANT | ENSECAG00000021273 | ENSECAT00000022601 |
| 28 | 34955841 | 34987054 | 31214 | | 34959831 | | G | | A | MODERATE | MISSENSE_VARIANT | TRIOBP | ENSECAT00000012534 |
| 28 | 34955841 | 34987054 | 31214 | | 34959831 | | G | | A | MODERATE | MISSENSE_VARIANT | TRIOBP | ENSECAT00000012539 |
| 28 | 34955841 | 34987054 | 31214 | | 34959968 | | C | | T | MODERATE | MISSENSE_VARIANT | TRIOBP | ENSECAT00000012534 |
| 28 | 34955841 | 34987054 | 31214 | | 34959968 | | C | | T | MODERATE | MISSENSE_VARIANT | TRIOBP | ENSECAT00000012539 |
| 28 | 34955841 | 34987054 | 31214 | | 34960466 | | A | | G | MODERATE | MISSENSE_VARIANT | TRIOBP | ENSECAT00000012534 |
| 28 | 34955841 | 34987054 | 31214 | | 34960466 | | A | | G | MODERATE | MISSENSE_VARIANT | TRIOBP | ENSECAT00000012539 |
| 28 | 34955841 | 34987054 | 31214 | | 34960716 | | C | | T | MODERATE | MISSENSE_VARIANT | TRIOBP | ENSECAT00000012534 |
| 28 | 34955841 | 34987054 | 31214 | | 34960716 | | C | | T | MODERATE | MISSENSE_VARIANT | TRIOBP | ENSECAT00000012539 |
| 28 | 34955841 | 34987054 | 31214 | | 34960773 | | T | | C | MODERATE | MISSENSE_VARIANT | TRIOBP | ENSECAT00000012534 |
| 28 | 34955841 | 34987054 | 31214 | | 34960773 | | T | | C | MODERATE | MISSENSE_VARIANT | TRIOBP | ENSECAT00000012539 |
| 28 | 34955841 | 34987054 | 31214 | | 34963441 | | T | | G | MODERATE | MISSENSE_VARIANT | TRIOBP | ENSECAT00000012534 |
| 28 | 34955841 | 34987054 | 31214 | | 34963441 | | T | | G | MODERATE | MISSENSE_VARIANT | TRIOBP | ENSECAT00000012539 |
| 28 | 34955841 | 34987054 | 31214 | | 34979790 | | G | | A | MODERATE | MISSENSE_VARIANT | TRIOBP | ENSECAT00000012534 |
| 28 | 34955841 | 34987054 | 31214 | | 34979790 | | G | | A | MODERATE | MISSENSE_VARIANT | TRIOBP | ENSECAT00000012539 |
| 28 | 34955841 | 34987054 | 31214 | | 34980697 | | C | | G | MODERATE | MISSENSE_VARIANT | TRIOBP | ENSECAT00000012534 |
| 28 | 34955841 | 34987054 | 31214 | | 34980697 | | C | | G | MODERATE | MISSENSE_VARIANT | TRIOBP | ENSECAT00000012539 |
| 28 | 34955841 | 34987054 | 31214 | | 34985838 | | C | | T | MODERATE | MISSENSE_VARIANT | TRIOBP | ENSECAT00000012534 |
| 28 | 34955841 | 34987054 | 31214 | | 34985838 | | C | | T | MODERATE | MISSENSE_VARIANT | TRIOBP | ENSECAT00000012539 |
| 28 | 34955841 | 34987054 | 31214 | | 34985844 | | A | | G | MODERATE | MISSENSE_VARIANT | TRIOBP | ENSECAT00000012534 |
| 28 | 34955841 | 34987054 | 31214 | | 34985844 | | A | | G | MODERATE | MISSENSE_VARIANT | TRIOBP | ENSECAT00000012539 |
| 28 | 34955841 | 34987054 | 31214 | | 34985877 | | G | | A | MODERATE | MISSENSE_VARIANT | TRIOBP | ENSECAT00000012534 |
| 28 | 34955841 | 34987054 | 31214 | | 34985877 | | G | | A | MODERATE | MISSENSE_VARIANT | TRIOBP | ENSECAT00000012539 |
|  |  |  |  | |  | |  | |  |  |  |  |  |
| Additional file 7. continued | | | | |  | |  | |  |  |  |  |  |
| chromosome | ROH start | ROH end | size | | position | | reference allele | | mutant allele | impact | type | gene | transcript |
| 28 | 35140321 | 35208630 | 68310 | | 35149332 | | A | | G | MODERATE | MISSENSE_VARIANT | SOX10 | ENSECAT00000025987 |
| 28 | 35140321 | 35208630 | 68310 | | 35157214 | | C | | G | MODERATE | MISSENSE_VARIANT | SOX10 | ENSECAT00000025987 |
