# Supplementary material for: Runs of homozygosity reveal signatures of positive selection for reproduction traits in breed and non-breed horses
Source: BMC Genomics. 2015 Oct 9;16:764. doi: 10.1186/s12864-015-1977-3 (PMC4600213; doi:10.1186/s12864-015-1977-3)
Supplement: Additional file 9: — Mutations with high or moderate effects in ROHs (50-SNP windows) of the four Hanoverians. The ROH position and size (EquCab2.70), the position of SNPs, their mutant allele, potential impact and type are shown. Impact estimations are derived from SNPEff predictions. (DOCX 15 kb) [file 12864_2015_1977_MOESM9_ESM.docx]

Additional file 9. Mutations with high or moderate effects in ROHs (50-SNP windows) of the four Hanoverians. The ROH position and size (EquCab2.70), the position of SNPs, their mutant allele, potential impact and type are shown. Impact estimations are derived from SNPEff predictions.

| Chromosome | ROH start | ROH end | Size | Position | Reference allele | Mutated allele | Impact | Type | Gene | Transcript |
| --- | --- | --- | --- | --- | --- | --- | --- | --- | --- | --- |
| 1 | 135530600 | 135573693 | 43094 | 135539894 | C | A | MODERATE | MISSENSE_VARIANT | DYX1C1 | ENSECAT00000011564 |
| 1 | 135530600 | 135573693 | 43094 | 135553628 | G | C | MODERATE | MISSENSE_VARIANT | ENSECAG00000004438 | ENSECAT00000004309 |
| 1 | 135530600 | 135573693 | 43094 | 135553829 | C | A | MODERATE | MISSENSE_VARIANT | ENSECAG00000004438 | ENSECAT00000004309 |
| 6 | 70162066 | 70181092 | 19027 | 70164923 | C | T | MODERATE | MISSENSE_VARIANT | CSAD | ENSECAT00000007643 |
